# Diversity and adaptation properties of actinobacteria associated with Tunisian stone ruins

**DOI:** 10.3389/fmicb.2022.997832

**Published:** 2022-12-13

**Authors:** Ilhem Saadouli, Ramona Marasco, Lassaad Mejri, Haytham Hamden, Meriem M’saad Guerfali, Panagiota Stathopoulou, Daniele Daffonchio, Ameur Cherif, Hadda-Imene Ouzari, George Tsiamis, Amor Mosbah

**Affiliations:** ^1^Laboratory of Microorganisms and Active Biomolecules, LMBA-LR03ES03, Faculty of Sciences of Tunis, University of Tunis El Manar, Tunis, Tunisia; ^2^Biological and Environmental Sciences and Engineering Division (BESE), King Abdullah University of Science and Technology (KAUST), Thuwal, Saudi Arabia; ^3^Laboratory “Energy and Matter for Development of Nuclear Sciences” (LR16CNSTN02), National Center for Nuclear Sciences and Technology, Sidi Thabet Technopark, Sidi Thabet, Tunisia; ^4^Laboratory of Biotechnology and Nuclear Technologies, LR16CNSTN02, National Centre of Nuclear Sciences and Technologies, Sidi Thabet, Tunisia; ^5^Laboratory of Systems Microbiology and Applied Genomics, Department of Sustainable Agriculture, University of Patras, Agrinio, Greece; ^6^BVBGR-LR11ES31, Higher Institute of Biotechnology Sidi Thabet, University of Manouba, Biotechpole Sidi Thabet, Ariana, Tunisia

**Keywords:** stone niches, Actinobacteria, oligotrophic environment, mineral solubilization, antimicrobial activities

## Abstract

Stone surface is a unique biological niche that may host a rich microbial diversity. The exploration of the biodiversity of the stone microbiome represents a major challenge and an opportunity to characterize new strains equipped with valuable biological activity. Here, we explored the diversity and adaptation strategies of total bacterial communities associated with Roman stone ruins in Tunisia by considering the effects of geo-climatic regions and stone geochemistry. Environmental 16S rRNA gene amplicon was performed on DNA extracted from stones samples collected in three different sampling sites in Tunisia, along an almost 400km aridity transect, encompassing Mediterranean, semiarid and arid climates. The library was sequenced on an Illumina MiSeq sequencing platform. The cultivable Actinobacteria were isolated from stones samples using the dilution plate technique. A total of 71 strains were isolated and identified based on 16S rRNA gene sequences. Cultivable actinobacteria were further investigated to evaluate the adaptative strategies adopted to survive in/on stones. Amplicon sequencing showed that stone ruins bacterial communities were consistently dominated by Cyanobacteria, followed by Proteobacteria and Actinobacteria along the aridity gradient. However, the relative abundance of the bacterial community components changed according to the geo-climatic origin. Stone geochemistry, particularly the availability of magnesium, chromium, and copper, also influenced the bacterial communities’ diversity. Cultivable actinobacteria were further investigated to evaluate the adaptative strategies adopted to survive in/on stones. All the cultivated bacteria belonged to the Actinobacteria class, and the most abundant genera were Streptomyces, Kocuria and Arthrobacter. They were able to tolerate high temperatures (up to 45°C) and salt accumulation, and they produced enzymes involved in nutrients’ solubilization, such as phosphatase, amylase, protease, chitinase, and cellulase. Actinobacteria members also had an important role in the co-occurrence interactions among bacteria, favoring the community interactome and stabilization. Our findings provide new insights into actinobacteria’s diversity, adaptation, and role within the microbiome associated with stone ruins.

## Introduction

In the planet’s harsh arid regions, the stone niche offers protection and relief from stresses such as solar UV radiation, desiccation, low nutrient availability, and extreme diel shifts in temperature (Wierzchos et al., 2012). A lithobiontic microbial community inhabits different portions of stones, including the up-surface in contact with air (epilithic community), underside surface in contact with soil (hypolithic community), and within the stone (endolithic community; Wierzchos et al., 2012). According to recent study, the stone microbiome on stone monuments is mainly dominated by Actinobacteria, Proteobacteria, Firmicutes, and Cyanobacteria (Marvasi et al., 2021; Ding et al., 2022). These communities form biofilms, or assemblages of microbes that stick to each other and to a surface that promotes community survival by retaining water and nutrients (Skipper et al., 2022). Like any ecological community, the members of these biofilms fulfill specific roles that help promote the survival of the entire community. The filaments of Actinobacteria and other filamentous microbes can grow and extend into the stone, facilitating not only the attachment of the microbiome to the stone, but also access to minerals within the stone (Ennis et al., 2021; Ding et al., 2022). In contrast, nonfilamentous microbes, such as various Proteobacteria and Firmicutes form aggregates that aid biofilm development and community cohesion (Ding et al., 2022). Autotrophic microorganisms of Cyanobacteria act as primary producers of the community and can provide the initial carbon sources to the stone surfaces, modify the surface characteristics and diversify the community from nutrient-poor to enrichment of heterotrophs on surfaces (Gu and Katayama, 2021).Climate and environmental conditions are the main driving factors that are responsible for the biological colonization of stone, especially for open-air sites (Zhang et al., 2021; Ding et al., 2022). Also the Geochemistry influences the microbial community structure, likely by impacting rates of microbial colonization and limiting access to minerals and nutrients required for growth (Louati et al., 2020; Ennis et al., 2021).

Actinobacteria from lithic materials have received research attention as they provide biological insights into the physiological mechanisms that allow them to survive and even thrive under prohibitive conditions (Meklat et al., 2011). Actinobacteria were frequently reported as the principal deteriorating forces of stone artifacts and buildings (Abdulla, 2009). Their ability to interact with minerals, metals, metalloids, and organic compounds through biomechanical and biochemical processes makes them ideally suited as biological weathering agents (Abdulla, 2009; Wang et al., 2022). Additionally, they are involved in the biogeochemical cycling of essential nutrients and metals because of their fundamental importance as decomposers of complex molecules. Genomic studies of actinobacteria isolated from stones have revealed such capacities, including, among others, genes for the synthesis of starvation inducible proteins, biofilm formation, or the protection and repair of DNA (Sghaier et al., 2016). Many actinobacteria produce a wide array of secondary metabolites (Lewin et al., 2016; van Bergeijk et al., 2020), such as siderophores and enzymes that enhance nutrient availability (Sathya et al., 2017). Furthermore, these microorganisms are sources of many bioactive metabolites and antimicrobial compounds (Rangseekaew and Pathom-aree, 2019). The multiple-stress-selecting conditions occurring in the lithic materials are considered to endow such microorganisms with unique metabolic traits to enhance their survival. So, they have been proposed as a source of novel metabolites for various potential applications (Sun et al., 2018). Members of the *Blastococcus*, *Geodermatophilus*, *Kocuria*, and *Modestobacter* genera are known to be resistant to desiccation, ionizing radiation, UV-light, and heavy metals (Sghaier et al., 2016; Sajjad et al., 2018; Sayed et al., 2020; Liu et al., 2022).

In this study, we have explored the bacterial community structure in stone materials recovered from ancient ruins collected from three distinct climate regions in Tunisia, encompassing the Mediterranean, semi-arid and arid regions. We hypothesized that (i) they are inhabited by diverse microbial communities, and within them, different actinobacteria are selected by the changing environmental and geoclimatic conditions, and (ii) that the cultivable actinobacteria are equipped with functional traits for the adaptation to the harsh conditions of the stones and potentially useful for biotechnological applications.

## Materials and methods

### Sampling sites, stones collection, and preparation

The samples used for this investigation were randomly collected from three different historic Roman stone ruins in Tunisia.

The selected Roman ruins are distributed along an almost 400 km aridity transect, encompassing Mediterranean (Dougga: 36°25′15.33″N, 9°13′2.08″E), semiarid (Haidra: 35°10′8.00″N, 8°47′24.00″E), and arid (Ksar Ghilane: 32°59′53.18″N, 9°38′26.45″E) climates (Table 1). The climate of the sites was defined by using the Wladimir Köppen climatic classification (Kottek et al., 2006) and the high-resolution KMZ for Google Earth available at http://koeppen-geiger.vu-wien.ac.at/present.htm. The three sites were classified as Csa (warm temperate), BSk (cold arid) and BWh (hot arid) (see Supplementary Figure S1). Data related to climate (max and min temperature and precipitation) were collected from climate knowledgeportal.worldbank.org, considering the period 1991–2020. Aridity values were obtained from the package R Envirem (Title and Bemmels, 2018) at the resolution of 1 km^2^ and mapped as described in (Marasco et al., 2022).

**Table 1:** Location and climate conditions of the three sampling sites in which stone ruins were collected.

Location	Ruin	N. of samples	Climate type	Rainfall(mm/year)	Max/min temperature (°C)	Altitude (m)
Dougga	Arch of Severus Alexander	3	Mediterranean	598	36/5	378
Haidra	Arch of Septimius Severus	3	Semi-arid	430	36/2	1,633
Ksar Ghilane	Small Fort Tisavar	3	Arid	126	39/6	224

Köppen-Geiger climatic classification map is reported in Supplementary Figure S1. Rainfall and temperature are reported as average values.

At each location, three areas of 1 m × 1 m were sampled, and further used as replicates. The distance between the replicates was approximately 5 m. From each replicate area, about 10 g of stone was collected aseptically from the stone ruins using a sterile rock hammer. The samples from each replicate were crushed in a sterile mortar and pestle, homogenized and the resulting powder was used for the isolation of Actinobacteria, as well as for geochemistry and high-throughput sequencing analysis.

### Geochemistry of stone and climatic parameters of ruins

Geochemical analysis was performed in the Centre of Water Researches and Technologies of Tunisia, using X-Ray diffraction (Zimmermann et al., 2015). The samples were pulverized and then dry at room temperature. Next, we have sieved at <100 μm. Then the samples were successively rinsed with a 0.1 mol L^−1^ HCl and NaOH solutions, to eliminate the most soluble fractions. After a subsequent filtration and a water wash, the obtained insoluble solid was stirred in a nitric acid (5. 10^−2^ mol L^−1^) solution to saturate the proton surface sites. The following elements were determined: Arsenic (As), Plomb (Pb), Cadmium (Cd), Chrome (Cr), Copper (Cu), Iron (Fe), Zinc (Zn), Calcium (Ca), Magnesium (Mg), and Sodium (Na). The table containing these data was normalized and used to create a resemblance matrix using the Euclidean distance in PRIMER (Gorley and Clarke, 2008). Canonical analysis of principal coordinates (CAP) was performed to test the significant difference in the stones’ geochemistry across the three climates. Significant differences in each element were further determined using the analysis of variance (ANOVA) test (*p* < 0.05).

### Total DNA extraction, PCR amplification, library preparation, and sequencing

Stone samples were aseptically smashed in powder by grinding with a sterile mortar and pestle. According to the manufacturer’s protocol, approximately 0.5 ± 0.05 grams of stone powder were used to extract total DNA using the PowerSoil DNA Isolation Kit (MoBio, United States). A fragment of approximately 460 bp belonging to the V3–V4 region of the bacterial 16S rRNA gene was amplified by PCR using the universal primer set U341FMiSeq, 5′-CCTACGGGRSGCAGCAG-3′, and 805RMiSeq, 5′-GACTACHVGGGTATCTAATCC-3′ (Klindworth et al., 2013). Amplification was performed using KAPA HiFi Hot-Start PCR Kit (Kapa Biosystems). Each 25 μl reaction contained 5 μl of KAPA HiFi Fidelity Buffer (5×), 0.7 μl of dNTPs solution (10 mM each), 0.7 μl of each primer solution (10 μM), 0.3 μl of KAPA HiFi Hot-Start DNA Polymerase solution (1 U/μl), 1 μl from the template DNA solution and 16.6 μl of sterile deionized water. The PCR protocol included an initial denaturation step at 95°C for 3 min, followed by 30 cycles of denaturation at 98°C for 20 s, annealing at 60°C for 15 s, and extension at 72°C for 45 s. The reaction was terminated with a final extension step at 72°C for 1 min. The appropriate negative and positive controls for each set of PCR reactions were also prepared. The approximately 550 bp amplification products (size increase due to the incorporation of the 50-mer Illumina primers) were electrophoresed on a 1.5% w/v agarose gel and visualized in Bio-Rad’s Gel Doc™ XR+ system. Positive PCR products were purified with a 20% PEG, 2.5 M NaCl solution, centrifuged at 14.000× g for 20 min, and the precipitate was washed twice with 125 μl of a 70% v/v ethanol solution and centrifuged at 14.000× g for 10 min as previously described (Ntougias et al., 2016). The dried precipitates were suspended in 15 μl of sterile deionized water, and the concentration was measured with a Quawell Q5000 micro-volume UV–Vis spectrophotometer.

The purified PCR products were diluted to a final concentration of 10 ng/μl and submitted to indexing PCR to incorporate the Illumina adapters (barcodes). Each sample was amplified with a unique combination of index primers during indexing PCR. Amplification was performed in 50 μl reactions using the KAPA HiFi Hot-Start PCR Kit. Each reaction contained 10 μl of KAPA HiFi Fidelity Buffer (5×), 1.5 μl of dNTPs solution (10 mM each), 5 μl of the forward index primer (10 μM), 5 μl of the reverse index primer (10 μM), 1 μl of KAPA HiFi Hot-Start DNA Polymerase (1 U/μl), 2 μl from the diluted PCR product (10 ng/μl), and 25.5 μl of sterile deionized water. The PCR program comprised an initial denaturation step at 95°C for 3 min, followed by eight cycles of denaturation at 95°C for 30 s, annealing at 55°C for 30 s, and extension at 72°C for 30 s. The reaction was terminated with a final extension step at 72°C for 5 min. The resulting amplicons were purified using Macherey-Nagel’s NucleoMag® NGS Clean-up and Size Selection kit (MACHEREY-NAGEL GmbH & Co, Düren, Germany) according to the manufacturer’s recommendations. Purified samples were suspended in 30 μl of sterile deionized water, and their concentration was measured with a Quawell Q5000 microvolume UV–Vis spectrophotometer (Quawell, San Jose, CA, United States). All samples were diluted to a final concentration of 8 nM and mixed equimolarly. The library was sequenced on an Illumina MiSeq sequencing platform by Macrogen (Korea). Raw reads have been deposited in the Short Reads Archive of NCBI under BioProject ID PRJNA847910.

### Analysis of amplicon sequences and bacterial diversity

Sequencing reads were de-multiplexed and converted to FASTQ. The Illumina adapters were removed using Illumina standard algorithms. Paired-end reads were assembled, trimmed by length, and corrected using the usearch-fastq_mergepairs option. Analysis of reads was performed using usearch v.10 (Edgar et al., 2011). The quality of the assembled sequences was further improved using the -fastq_filter, followed by finding unique read sequences and abundances by using the -fastx_uniques option. Sequences were clustered into operational taxonomic units (OTUs) using the cluster_otus command (Edgar, 2013). Chimeras were removed using the -unoise3 option (Edgar, 2016). Taxonomy was assigned using the SILVA 16S rRNA gene database (release 128; Quast et al., 2013). OTUs classified as chloroplasts (7 OTUs, 34,956 reads) were removed, and the rarefaction curves were generated (Supplementary Figure S2).

Alpha-diversity indices (richness, Shannon diversity, and Simpson diversity) were calculated in R using the Phyloseq package, and significant differences across the three climatic groups were determined using the analysis of variance (ANOVA) test (*p* < 0.05) in GraphPad (Prism 9.2). Between-sample beta-diversity was evaluated by computing Bray–Curtis (BC) similarity (Bray and Curtis, 1957) on log-transformed data starting from all the bacterial OTUs (*n* = 99) and the OTUs belonging to Actinobacteria (*n* = 37). Principal coordinates analysis (PCoA; Gower, 1998) was performed, along with the distant-based test PERMDISP, to test the homogeneity of multivariate dispersion for the factor “Climatic region” (3 variables: the Mediterranean, semiarid, and arid) before running permutational analysis of variance (PERMANOVA) in Primer (Anderson, 2001). The two BC-similarity matrices (bacteria and Actinobacteria) were used to perform decay relationships with geochemistry (based on Euclidean distance), average rainfall per year, and altitude. Distance-based multivariate analysis for a linear model (DistLM; Anderson, 2004) with Akaike information criterion (AIC) (Bozdogan, 1987) was used to determine which significant geochemical variables explain the observed BC similarities among the stones’ bacterial communities. The contribution of each geochemical variable (i.e., stone elements) was assessed by running marginal and sequential tests. Two-way hierarchical clustering analysis was run with *latticeExtra* in R and used to identify which OTUs are the most important for sample clustering; only OTUs comprised greater than 0.4% of relative abundance were displayed. Shared (i.e., core microbiome) and specific bacterial OTUs of stones across the three climates were defined using Venn-diagram analysis with R. The linear discriminant analysis (LDA) effect size (LEfSe) analysis was employed to identify the bacterial groups that were significantly differentiated among sample types. The LDA was performed using a one-against-all strategy, an alpha significance level of 0.05, and an effect-size threshold of 3 for all distinctive taxa (Segata et al., 2011). A correlation network between the bacterial OTUs was built by calculating all pairwise Pearson correlation coefficients among them in Conet (Faust and Raes, 2016). The co-occurrence network was visualized with Gephi (Bastian et al., 2009) and default parameters were used to identify modules of soil taxa strongly interacting with each other. Categories of interaction were computed and classified based on the taxonomic affiliation of nodes involved.

### Isolation and identification of cultivable actinobacteria

For isolation of actinobacteria, 1 g of smashed rock was used following the serial dilution plate culture technique using Luedemann medium (Luedemann, 1968) and R2A (Difco). These media were successfully used in the past to isolate Actinobacteria from rocks/stones (Essoussi et al., 2012; Hezbri et al., 2016). Plates were incubated at 30°C for 2 weeks in an incubator. Morphologically distinct colonies were picked from mixed colonies and sub-cultured onto fresh medium to obtain pure cultures. The selected isolates were stored in 20% glycerol tubes and kept at −80°C for long-term storage.

The bacterial collection was dereplicated by fingerprinting analysis of the rRNA 16S-23S intergenic transcribed spacer (ITS) region. ITS-PCR amplifications were carried out using the universal primers, ITSF (5′-GTCGTAACAAGGTAGCCGTA-3′) and ITSR (5′-CAAGGCATCCACCGT-3′; García-Martínez et al., 1999). The ITS-PCR amplification consisted of 1 × PCR reaction buffer, 4 μl MgCl_2_ (1.5 mM), 0.25 μl dNTP (0.2 mM), 0.3 μl of each primer (0.5 μM), 0.22 μl Taq-polymerase (5 U/μl) and 100 ng of DNA extracted from single colonies. The total volume was adjusted to 25 μl. Amplification parameters were as follows: initial denaturation at 95°C for 5 min, followed by 35 cycles at 95°C for 30 s, 57°C for 35 s, 72°C for 2 min, with a final extension step of 10 min at 72°C.

One or more strains for each ITS group have been selected for subsequent identification using 16S rRNA gene sequencing. Amplification of the 16S rRNA gene was carried out with universal bacterial primers 27F (5′-AGAGTTTGATCMTGGCTCAG-3′) and 1492R (5′-CTACGGCTACCTTGTTACGA-3′; Chen et al., 2010). The PCR was run for 35 cycles (denaturation at 95°C and 30 s, annealing at 55°C and 35 s, extension at 72°C and 2 min) with one initial denaturation step (95°C, 5 min) and a final extension step (72°C, 10 min). The ITS and 16S rRNA gene PCR amplification products were migrated on 2% and 1.5% agarose gels in 0.5× Tris-borate-EDTA buffer and stained with ethidium bromide (Sigma-Aldrich).

The 16S rRNA sequencing was carried out using the BigDye Terminator v3.1 Cycle Sequencing Kit and the ABI 3130 sequence analyzer. The nucleotide sequences of the 16S rRNA gene obtained were analyzed by BLAST and compared with those available at the National Centre for Biotechnology Information (NCBI) database^1^ and Ribosomal Database Project (RDP) and submitted to GenBank. Phylogenetic analysis of the 16S rRNA gene sequences was conducted with Molecular Evolutionary Genetics Analysis (MEGA) software, version 11 (Tamura et al., 2021). The bacteria not belonging to the phylum of Actinobacteria were eliminated from the analysis The tree was constructed using the neighbor-joining method (Saitou and Nei, 1987). The topologies of the neighbor-joining phylogenetic trees were evaluated in bootstrap analyses of 1,000 replicates. The 16S rRNA gene sequences of isolates were deposited in the GenBank database under the accession numbers ON713902-ON713972.

### The characterization *in vitro* of abiotic stress tolerance and nutrient acquisition abilities

The 71 actinobacterial strains identified were screened *in vitro* to test their adaptation to oligotrophic and extreme rock environments. The ability to release phosphate from insoluble inorganic and organic P forms was tested; the strains were cultured under shaking (120 rpm) for 7 days at 30°C in NBRIP (National Botanical Research Institute’s phosphate growth medium) and PSM (phytase−screening medium) broths, containing Ca − phosphate (Ca_3_(PO_4_)_2_) and Na − phytate (C_6_H_18_O_24_P_6_·xNa + ·yH_2_O) as sole phosphate (P) sources, respectively(Jorquera et al., 2008). Non-inoculated broths served as controls. After incubation, the cultures were harvested by centrifugation at 10,000 rpm for 10 min. The released orthophosphate (P) in the supernatant was measured by the method reported by Bae and colleagues (Bae et al., 1999) and expressed as ug/ml. Nitrogen fixation was determined using a nitrogen-free medium (NFM). Isolates were incubated for 7 days, and bacterial growth was qualitatively checked at the end of incubation (Grobelak et al., 2015). For estimation of ammonia production, bacterial strains were grown in peptone water broth for 7 days at 30°C ± 2°C. One ml of culture supernatant was mixed with 1 ml of Nessler reagent, and this mixture was brought to 10 ml by adding ammonia-free distilled water. The development of the brown-yellow color indicates ammonia production, and its optical density was measured at 450 nm using a spectrophotometer (Demutskaya and Kalinichenko, 2010). The concentration of ammonia was estimated by using the standard curve of ammonium sulfate in the range of 0.1–100 μg/ml. The ability of Actinobacteria to produce IAA was assessed as described by (Patten and Glick, 2002). Bacterial cells were grown in tryptophan supplemented medium at 30°C for 7 days of cell harvesting (10,000 rpm for 10 min), followed by the addition of 2 ml of Salkowski’s reagent. The appearance of pink color indicated IAA synthesis after 25 min. OD was monitored at 535 nm using a Spectrophotometer (UV 3000 spectrophotometer). Commercial IAA (Sigma Aldrich) was used as standard. Bacterial isolates were assayed for siderophores production on the Chrome Azurol S agar medium (Sigma, Ltd.) following the protocol described by (Schwyn and Neilands, 1987). Strains grown in the iron-free medium are deposited as a disk on the surface of CAS agar. After 15 min, the development of a yellow-orange halo around the growth was considered positive for siderophore production. Resistance to salt was assessed by adding 5, 10, and 15% NaCl to the culture media and incubating the plates at 30°C for 7 days. The ability to grow at 45°C and 50°C was checked in Luedman media by incubation at the indicated temperatures for 7 days (Elsheikh, 1992). The presence/absence of growth was evaluated for salinity and temperature tests at the end of incubation.

### Extracellular enzyme production

The actinobacterial isolates were screened for the capacity to produce extracellular enzymes, namely alkaline phosphatase, amylase, protease, cellulase and chitinase. Bacterial cultures were grown at 30°C under agitation in 50 ml flasks for alkaline phosphatase assay. The cultures were centrifuged at 4000 rpm for 15 min. For the assay, 10 μl of p-NPP (5 mM) and 100 μl of Diethanolamine buffer (1 M) at pH 9.8 containing MgCl_2_ (0.50 mM) were added to 100 μl of each supernatant, which was previously plated in a 96-well plate. After incubation at 37°C for 30 min, the reaction was stopped by adding 100 μl of NaOH (1 M) solution. Alkaline phosphatase activity is determined by measuring the formation of para-nitrophenolate at 405 nm and expressed as Units/ml (Yu Plisova et al., 2005). Qualitative estimation of amylase production was performed on the starch agar plate by starch hydrolysis assay (Raj et al., 2009). Strains were spot inoculated on starch agar plates. After incubation at 30°C ± 2°C for 7 days, plates were flooded with Gram’s iodine (Gram’s iodine—250 mg iodine crystals added to 2.5 mg potassium iodide solution and 125 ml of water). A deep blue colored starch-iodine complex is produced upon adding an iodine solution. The amylase producers would display a clearance zone, and no blue color would be formed surrounding the zone. To detect proteolytic activity, the actinobacteria isolates were spot inoculated on skim milk agar media and then incubated at 30°C for 7 days. Positive isolates were confirmed by clear areas around the colonies (Chang et al., 2009). Qualitative estimation of cellulase was done by spot inoculating actinobacterial strain on basal agar medium containing carboxymethyl cellulose (CMC; 0.5% w/v) as the sole carbon source. After incubation at 30°C ± 2°C for 7 days, all the plates were stained with 1% (w/v) of Congo-red solution for 15 min and discolored with 1 M NaCl for 15 min (Kasana et al., 2008). The zone of degradation around bacterial colonies indicated positivity to cellulase production. Chitinase production was assessed by spot inoculating actinobacteria isolates on M9 minimal salt agar plates containing 1% w/v colloidal chitin and 1.2 g yeast extract, followed by incubation for 7 days at 30°C ± 2°C. The development of a clearance zone around the colonies was considered positive for chitinase production (Hsu and Lockwood, 1975).

### Antimicrobial activity assay

Antimicrobial activities were examined *in vitro* against standard strains of bacterial and fungal pathogens that include (i) Gram-positive bacteria, such as *Staphylococcus aureus*, *Bacillus cereus* NR074540.1, *Enterococcus faecalis* MK584170, (ii) Gram-negative bacteria, such as *Escherichia fergusonii* MK584171, *Pseudomonas aeruginosa* MK584172, and *Salmonella enterica* MK584173, (iii) medically important dermatophytic fungi, such as *Candida albicans* MK599152, *Candida metapsilosis* MK599150, *Candida parapsilosis* MK599151, and (iv) phytopathogenic fungi, such as *Fusarium oxysporum*, *Penicillium expansum*, *Aspergillus flavus* and *Aspergillus niger.* All strains were obtained from the microbial collections of the Laboratory of Microorganisms and Active Biomolecules (LMBA), Faculty of Sciences of Tunis. The Agar Disc Technique determined the antimicrobial activity of actinobacterial cultures (Tortorano et al., 1979). Each actinomycete strain is streaked on the surface of the Luedman medium. After incubation at 30°C for 7 days, 6 mm diameter agar cylinders were removed and placed in a Petri dish containing a culture of the test organisms that were grown on Muller Hinton medium (bacteria), on Potato Dextrose agar (PDA; fungi) and Sabouraud Dextrose agar (yeast). The Petri dishes are placed at 4°C for 1 h to allow diffusion of the active substances. Then, they are kept for incubation for 24 h at 37°C for bacterial and for 48 h at 28°C for fungal strains. The antimicrobial activity was determined by measuring the size of the inhibition zone.

## Results

### Environmental conditions of Roman ruins and geochemistry of their stones

**Figure 1:**
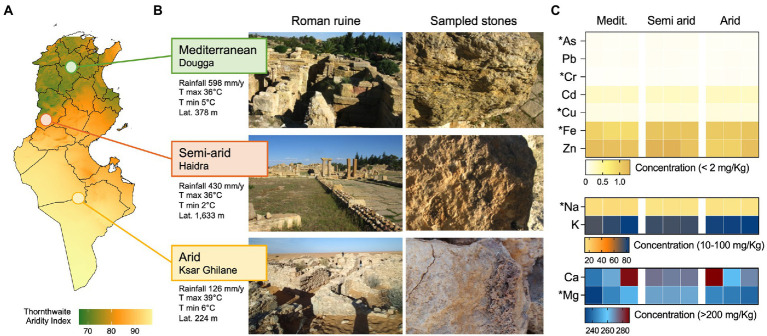
Ruins and climatic conditions along the aridity transect. **(A)** Map showing level of aridity in Tunisia expressed as Thornthwaite aridity index; the three sampling locations, namely Dougga, Haidra, and Ksar Ghilane, are indicated in the map and are categorized as Mediterranean, semiarid, and arid climates, respectively. **(B)** Representative images of the ruins and sampled stones in the three heritage sites. Climatic conditions of the three sites are also reported as average rainfall (mm/year), average max and min temperature (°C), and altitude (m). **(C)** The heat map reports the concentration (mg/kg) of stone elements along the aridity transect, namely the Mediterranean, semiarid, and arid climates. The elements are divided based on concentration ranges (<2 mg/kg, 10–100 mg/kg, and > 200 mg/kg) and their content is shown for each of the three replicates collected from each site.

We analyzed three heritage sites formed by Roman settlements in Tunisia. The three regions differ mainly in the amounts of rainfall received per year, from an average of 598 mm/year in the Mediterranean to 126 mm/year in the Saharan Ksar Ghilane, and for their altitude (224 m, 378 m and 1,633 m in Ksar Ghilane, Dougga, and Haidra, respectively; Figures 1A,B). The ruin stones sampled were analyzed for their element contents (Figure 1C). They are dominated by Mg and Ca, followed by K and Na and several minor elements, such as Zn, Fe and Cd. However, the concentration/distribution of these elements in the stones determined three different groups (CAP, delta_1^2: 0.97522, *p* = 0.023; Supplementary Figure S3A), which diversity is mainly explained by As, K, Fe, Na and Mg concentrations (Person *r* > 0.8; Supplementary Figure S3B).

### Diversity, structure, and composition of bacterial communities associated with ruin stones

**Figure 2:**
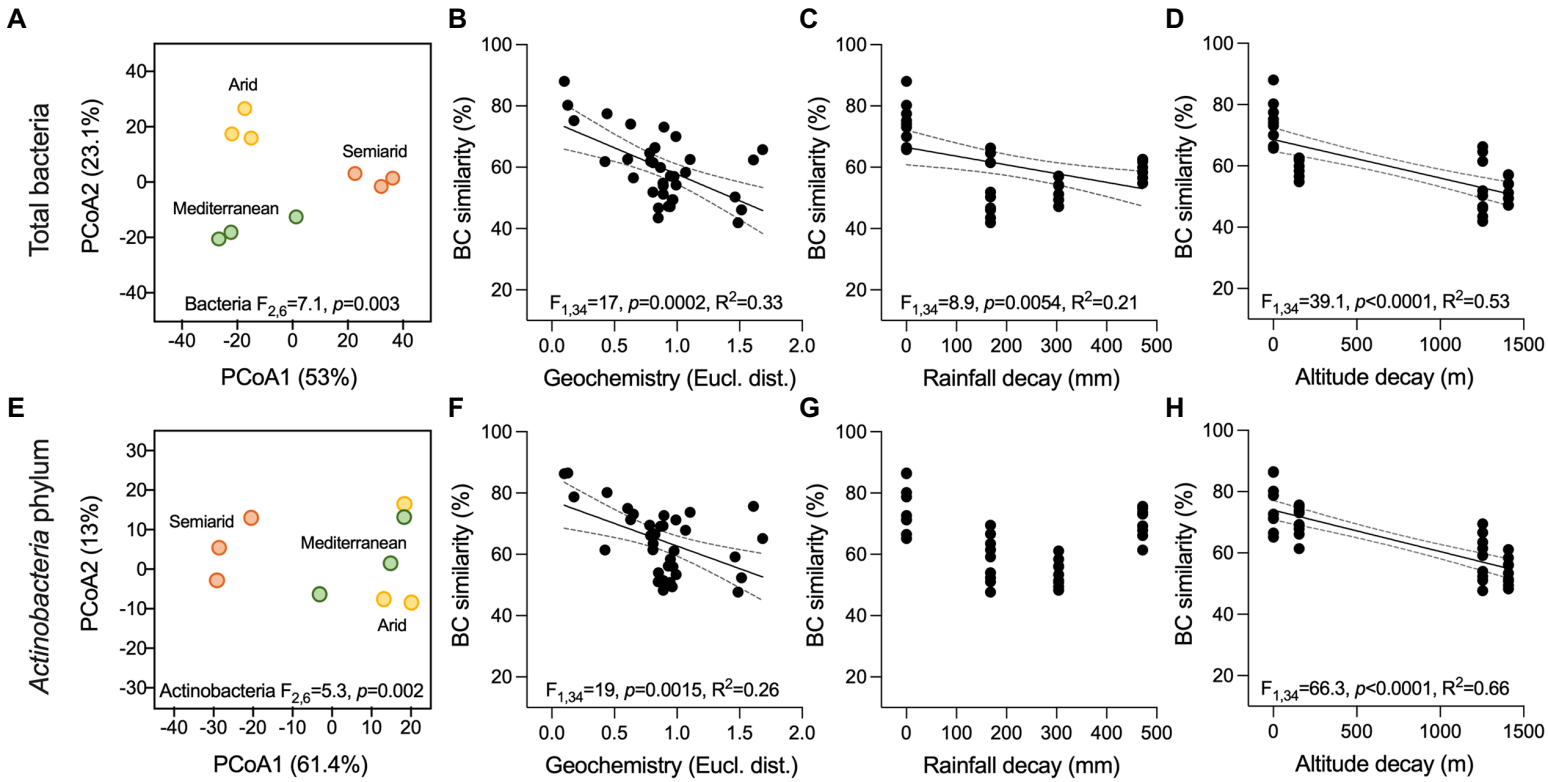
Beta-diversity of bacterial communities associated with the ruin stones along the climatic transect. **(A,E)** Principal coordinate analysis (PCoA) of the total bacterial communities and members of the *Actinobacteria* phylum associated with the stones, respectively. Results of PERMANOVA are reported in the graphs. **(B–D, F–H)** Bray Curtis (BC) similarity distance decay in function of **(C,F)** stone geochemical conditions (Euclidean distance), **(D,G)** average precipitations (mm/years), and **(E,H)** altitude (m) in the total bacterial communities and members of the *Actinobacteria* phylum, respectively. Black lines indicate significant linear regressions, and gray-dashed lines the 95% confidence bands; *value of p*s and R^2^ are reported in the graphs. Non-significant regression lines (*p* > 0.05) are not showed.

The bacterial community associated with stones from three ruins was investigated by 16S rRNA gene amplicon sequencing. After quality filtering, 341,669 reads (average, 37,963 ± 5,060 reads per sample) and 99 bacterial operational taxonomic units (OTUs) at a 97% sequence similarity were obtained. PCoA, based on the BC similarity distance metric, revealed a clustering of the bacterial communities according to the different climates along the aridity transect (PERMANOVA: F_2,6_ = 7.1, *p* = 0.003; Figure 2A; all pairwise comparisons, *p* < 0.05). The stone bacterial communities were differentiated along the two PCoA axes (53% and 23.1% of the total variance, respectively) with a cross-validation of 100% for the samples within each compartment (CAP). Besides using predefined climate categories (Mediterranean, semiarid, and arid), the effect of the ruins’ environmental contest (i.e., precipitation and altitude) and stones’ geochemical composition on the bacterial diversity was also evaluated. Decay analysis showed a significant decrease in bacterial BC similarity among samples for stone geochemistry (R^2^ = 0.33, *p* = 0.0002; Figure 2B), average annual rainfall (R^2^ = 0.21, *p* = 0.0054; Figure 2C) and altitude (R^2^ = 0.53, *p* < 0.0001; Figure 2D). Such decline in bacterial similarity had a different rate of decay (ANCOVA F_1,102_ = 29.7, *p* < 0.0001), with the altitude driving the highest decay (slope, −42.5), followed by stone geochemistry (slope, −17.3) and rainfall (slope, −0.029). Within the geochemistry, the DistLM marginal test indicated As, Mg and Na as the only significant variables influencing the bacterial distribution pattern (Table 2). Using the sequential test, only Mg was included (AICc = 59.923, R^2^ = 0.48).

**Table 2:** Correlation between physicochemical factors and the structure of bacterial and Actinobacteria communities associated with stones according to the Distance-based Linear Model (DistLM) marginal test that considers every single element and their contribution to explain the total variability.

Group	Variable	SS (trace)	Pseudo-F	*p*-value	Prop.
Bacteria (99 OTUs)	Arsenic*	2485.1	3.8928	0.021	0.35737
	Cadmium	1048.3	1.2426	0.295	0.15075
	Calcium	729.65	0.82058	0.513	0.10493
	Chrome	1382.4	1.7369	0.154	0.1988
	Copper	1464.8	1.8679	0.151	0.21064
	Iron	1,593	2.08	0.116	0.22907
	Magnesium*	3354.1	6.522	0.001	0.48233
	Plomb	441.15	0.47415	0.766	0.063438
	Potassium	1,284	1.5852	0.176	0.18464
	Sodium*	1998.8	2.8237	0.039	0.28744
	Zinc	1382.2	1.7365	0.143	0.19876
Actinobacteria (37 OTUs)	Arsenic*	2120.8	4.3964	0.026	0.38577
	Cadmium	457.21	0.63496	0.616	0.083165
	Calcium	461.12	0.64089	0.614	0.083877
	Chrome	363.74	0.49596	0.738	0.066163
	Copper	528.54	0.74456	0.492	0.09614
	Iron	769.13	1.1386	0.307	0.1399
	Magnesium*	3031.3	8.6037	0.002	0.55139
	Plomb	464.36	0.64581	0.571	0.084466
	Potassium	1,104	1.7589	0.165	0.20081
	Sodium	910.03	1.3886	0.23	0.16553
	Zinc	1228.6	2.0146	0.128	0.22349

Star (*) indicates a variable statistically significant, *p*-value < 0.05. Prop., proportion of total variation explained by the variable(s). The dataset is available in Supplementary Table S1.

**Figure 3:**
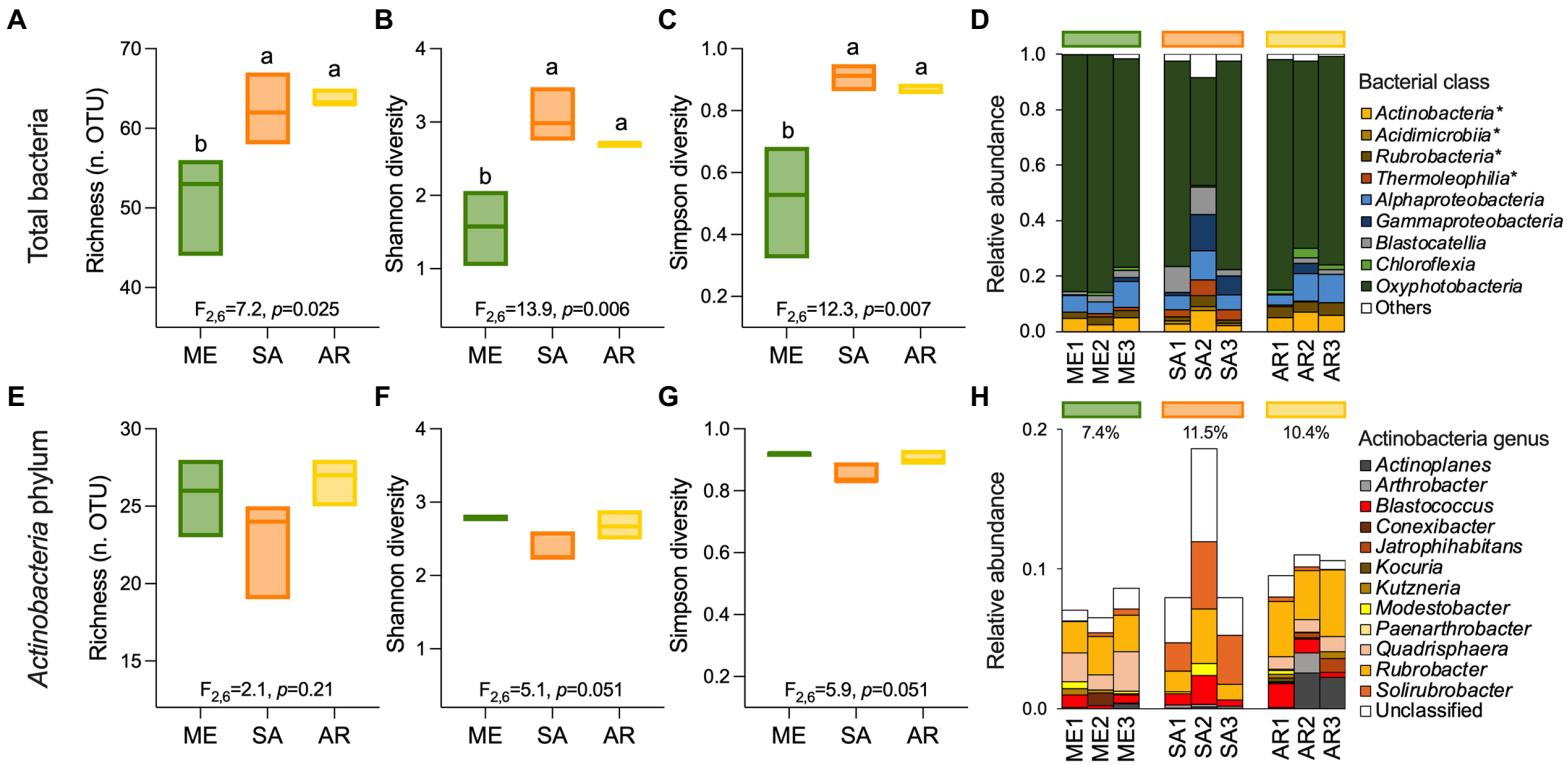
Alpha-diversity and composition of ruin stones’ bacterial communities. **(A,E)** Richness, **(B,F)** Shannon diversity, and **(C,G)** Simpson diversity indices for total bacterial communities (99 OTUs) and members of *Actinobacteria* phylum (37 OTUs) associated with the stones across the aridity transect. **(D)** Relative abundance of bacterial classes. Star (*) indicates classes belonging to the *Actinobacteria* phylum and “others” the low abundant groups (relative abundance <1% and non-belonging to Actinobacteria). **(H)** Relative abundance of genera within the *Actinobacteria* phylum.

**Figure 4:**
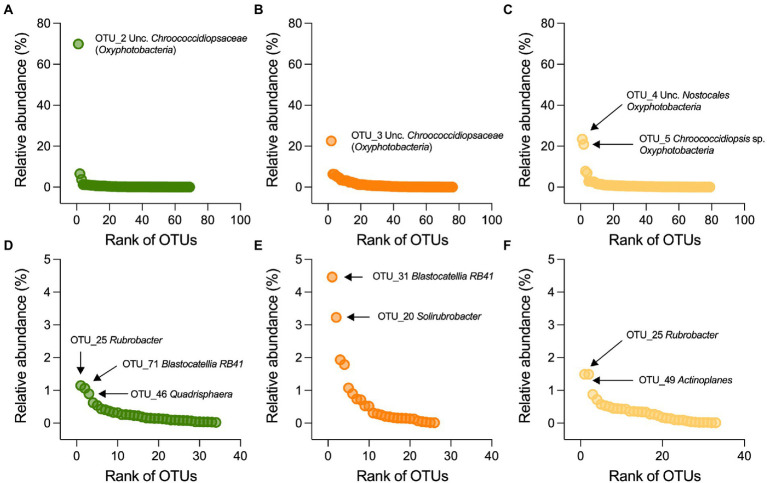
Distribution and taxonomic classification of dominant OTUs. Rank-abundance plots for **(A–C)** bacterial and (**D**–**F**) actinobacterial OTUs associated with stone from the Mediterranean (green), semiarid (orange) and arid (yellow) ruins; the OTUs are ranked on the x-axis based on their relative abundance (y-axis), from the most abundant (left side) to the lowest abundant (right side). Taxonomic affiliation of the dominant OTUs is also reported.

In the stone bacterial communities, richness (number of observed OTUs), Shannon diversity and Simpson diversity were significantly lower in the Mediterranean ruins (ANOVA: F_2,6_ = 7.2, *p* = 0.025, F_2,6_ = 13.9, *p* = 0.006 and F_2,6_ = 12.3, *p* = 0.007, respectively; Figures 2A–C). While alpha diversity indices decay showed positive relationships with geochemistry distance (i.e., the more stones’ geochemistry differs, the more the alpha diversity indices differ), they did not have a relationship with altitude. Only richness was negatively related to precipitation (Supplementary Table S2). Irrespective of the climate type, the stone bacterial communities were dominated by few abundant OTUs (7, 22 and 15 OTUs in the Mediterranean, semiarid and arid climates, respectively), followed by a tail of rare OTUs (relative abundance < 1%; range, 53–64 OTUs; Figure 4A-C). The dominant OTUs were different across the three locations, but all belong to the *Nostocales* order within the *Oxyphotobacteria* class of *Cyanobacteria* (Figure 4A-C) and consistently occupy at least 23% of the total relative abundance. Considering the overall taxonomical composition of the communities, *Oxyphotobacteria* were confirmed as the most abundant bacteria in the three stone groups (average 74.2% of relative abundance; Figure 3D). While *Nostocales* were found across all samples but with differential distribution, members of *Leptolyngbyales* and *Thermosynechococcales* were mainly detected in the ruins of the semiarid region. The bacterial communities were further composed by *Actinobacteria* (4.7% *Actinobacteria*, 2.9% *Rubrobacteria,* 1.6% *Thermoleophilia* and 0.5% *Acidimicrobiia*), *Proteobacteria* (6.9% *Alphaproteobacteria* and 2.8% *Gammaproteobacteria*), *Acidobacteria* (3.3% *Blastocatellia*) and *Chloroflexi* (1.1% *Chloroflexia*; Figure 3D). Notably, only *Thermoleophilia*, *Acidimicrobiia* and *Chloroflexia* showed a relative differential abundance across climates (Supplementary Figure S4).

### Diversity, structure, and composition of members affiliated to the *Actinobacteria* phylum

Of the 99 bacterial OTUs, 37 were taxonomically identified within the *Actinobacteria* phylum. These OTUs had medium-low relative abundance (0.02%–4.5%, Supplementary Figures 3E–F). They were differentially distributed across the three climates, creating distinct actinobacterial communities (PERMANOVA: F_2,6_ = 5.3, *p* = 0.002). The samples from the semiarid climate form a clear separate cluster in the ordination space of PCoA (Figure 2E). The actinobacterial BC similarity was mainly related with site altitude (R^2^ = 0.66, *p* < 0.0001) and variation in stone elements’ content (R^2^ = 0.26, *p* < 0.0015) but not with the average annual rainfall decay (Figures 2F–H). DistLM showed that As and Mg remain the significant element explaining the difference observed (Table 2).

Contrary to the total community, the alpha-diversity of members within the Actinobacteria phylum (richness, Shannon diversity, and Simpson diversity Figure 3A-E and Figure 3B-F) did not vary along the transect (ANOVA: F_2,6_ = 2.1, *p* = 0.21, F2,6 = 5.1, *p* = 0.051, and F_2,6_ = 5.9, *p* = 0.051; Figures 3E–G), indicating that this component of the bacterial community vary across different climates in term of composition (Figures 2E, 3H), but maintains a consistent structure (richness) with uneven distribution (low Shannon diversity and Simpson diversity). From taxonomy, it was evident that few genera composed the actinobacterial communities (Figure 3H): among the most abundant, we found *Rubrobacter* (3%), *Solirubrobacter* (1.3%) and several actinobacteria unclassified at the genus level (2.1%), followed by genera with relative abundance below 1%, including (in decreasing order) *Quadrisphaera*, *Blastococcus*, *Actinoplanes*, *Arthrobacter*, *Modestobacter*, *Kutzneria*, *Jatrophihabitans*, *Conexibacter*, *Kocuria* and *Paenarthrobacter.* While in all the stones *Rubrobacter* genus occupied an important portion of the community, stones from the semiarid region were preferentially colonized by *Solirubrobacter* and unclassified actinobacteria, those from the arid region by *Actinoplanes*, and those from the Mediterranean region by *Quadrisphaera* (Figure 3H; Figure 4E-F).

### Distribution of bacterial OTUs associated with ruin stones from different climate conditions

**Figure 5:**
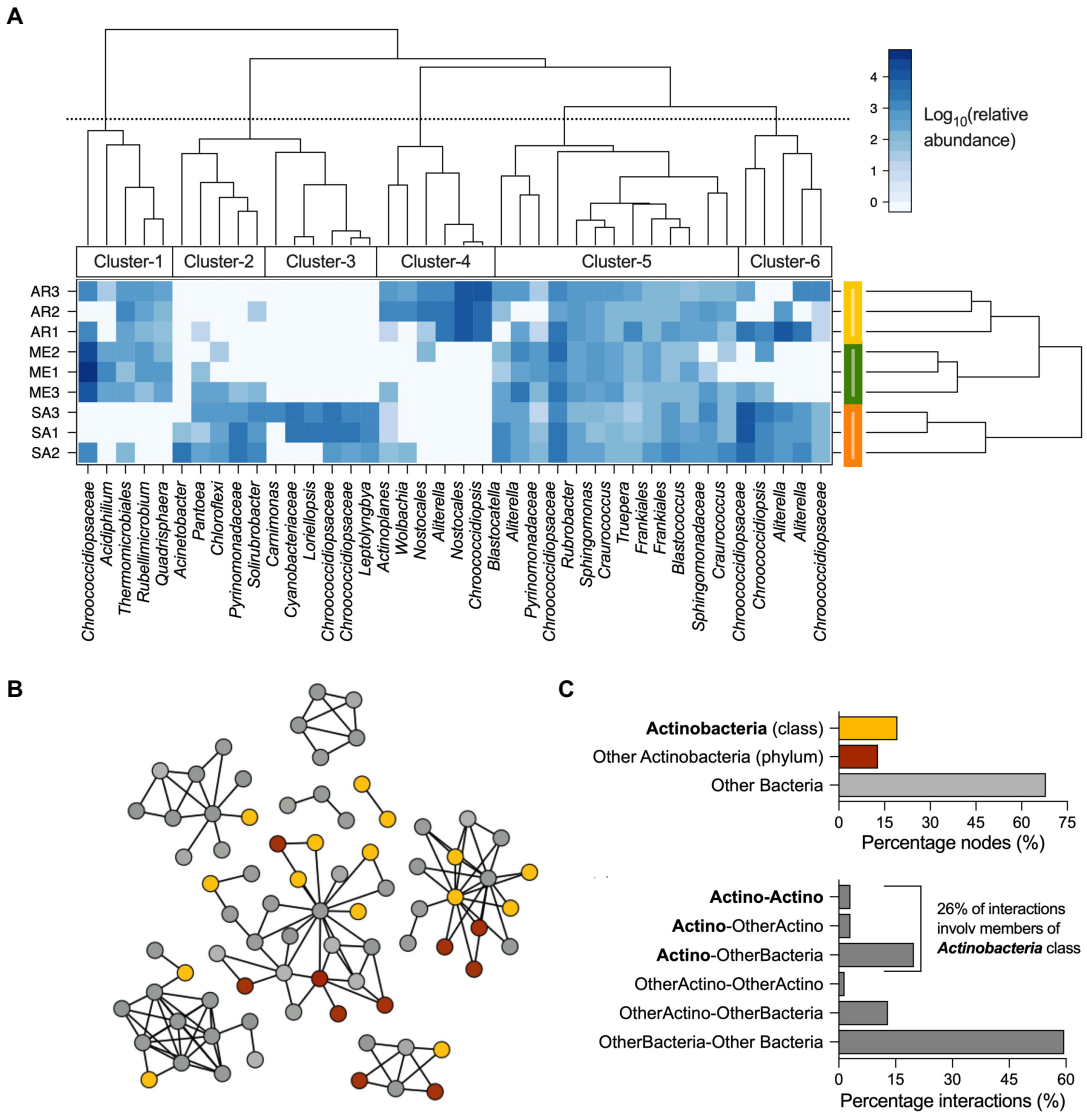
Distribution and interaction of bacteria associated with stones across the three climates. **(A)** Two-way cluster analysis of stone communities; most abundant bacterial OTUs (>0.4% relative abundance) are reported and how they are grouped in clusters based on their presence in the different climatic regions is indicated. **(B)** Co-occurrence correlation network of the bacterial OTUs associated with stones from Tunisian Roman ruins. Diagram of co-occurrence bacterial network with nodes colored by their taxonomic affiliation. **(C)** Classification of interactions (considering both negative and positive) based on the taxonomic affiliation of nodes as members of Actinobacteria class (“Actino”), members of Actinobacteria phylum different than Actinobacteria class (“other Actino”), other bacteria (all the remining taxonomic groups; “bacteria”); the possible interactions are “Actino-Actino,” “Actino-OtherActino,” “Actino-Bacteria,” and “OtherActino-Bacteria.”

To understand which bacterial OTUs were responsible for the differences among ruins (Figure 2A), a two-way cluster and LEfSe analyses were performed (Figures 4A; Supplementary Figure S5, respectively). The cluster analysis identified six clusters of OTUs that showed variable differential abundance across climatic groups (Figure 5A; see Supplementary Table S3 for taxonomic details). Cluster-5 was formed by 13 abundant OTUs consistently detected in all the samples, independently from their origin, including members of *Alphaproteobacteria* (4 OTUs), *Actinobacteria* (3 OTUs), *Blastocatellia* (2 OTUs), *Oxyphotobacteria* (2 OTUs), *Deinococci* (1 OTU) and *Rubrobacteria* (1 OTU) classes; cluster-2 (5 OTUs), cluster-3 (6 OTUs) and cluster-6 (5 OTUs) were mainly associated with stones from semiarid climate and were dominated by *Oxyphotobacteria* (10 OTUs), followed by *Gammaproteobacteria* (3 OTUs), *Blastocatellia, Thermoleophilia* and Chloroflexi (1 OTU each); cluster-1 (5 OTUs) was shared among rocks from the Mediterranean and arid climates and was dominated by *Alphaproteobacteria* (2 OTUs), *Actinobacteria, Chloroflexia* and *Oxyphotobacteria* classes; cluster-3 (6 OTUs) dominated the stones’ community from arid climate, with members of *Oxyphotobacteria* (4 OTUs) *Actinobacteria* and *Alphaproteobacteria*. The LEfSe analysis identified a total of 18 OTUs as the bacterial signature of stones from different climates (Supplementary Figure S5), with five as signature of the Mediterranean (*Kineosporiales*, *Quadrisphaera* and *Acidiphilium*), 12 of semiarid (members of *Verrrucomicrobia* phylum, unclassified *Oceanospirillales*, *Nitrincolaceae*, and *Neptuniibacter* sp.) and one of arid (*Blastococcus* sp.). The distribution of bacterial OTUs was further investigated by evaluating their presence/absence across samples to define shared (i.e., bacteria of the core microbiome) and those specific to climatic groups (Venn Diagram). The OTUs present in all the samples were 47% and accounted for 58.5% of the relative abundance. Those climate-specific were 22% (2, 12% and 8% in the Mediterranean, semiarid and arid, respectively) and accounted only for 26.9% of relative abundance (0.2%, 8.7%, and 18%, respectively). The remaining OTUs were equally distributed among the three combinations of climate pairs (i.e., Mediterranean-semiarid, Mediterranean-arid, and semiarid-arid).

We finally built a co-occurrence network between the members of the bacterial communities associated with the stones to evaluate their capacity to interact and their role within the microbial interactome (Figures 5B–C). As showed in Figures 5B, 78 on 99 OTUs were included in the co-occurrence network, forming 131 co-occurrence (126 and 5 positive and negative interactions, respectively). Among the interacting nodes, 25 belong to the *Actinobacteria* phylum, including *Acidimicrobiia* (1 node), *Actinobacteria* (15 nodes), *Rubrobacteria* (5 nodes) and *Thermoleophilia* (4 nodes) classes (Figure 5C), while the remaining 53 nodes were identified as *Blastocatellia* (3 nodes), *Bacteroidia* (3 nodes), *Chloroflexi* (6 nodes), *Oxyphotobacteria* (19 nodes), *Alpha* and *Gamma*-*proteobacteria* (20 nodes), *Verrucomicrobiae*, *Deinococci* and *Bacilli* (1 node each). It is important to note that Actinobacteria were involved in 40% of the interactions, with 26% of them acted by members of *Actinobacteria* class (Figure 5D), indicating that this group of bacteria represent an important component in the stability of stone bacterial communities.

### Isolation and phylogenetic analysis of culturable actinobacteria from stones

**Figure 6:**
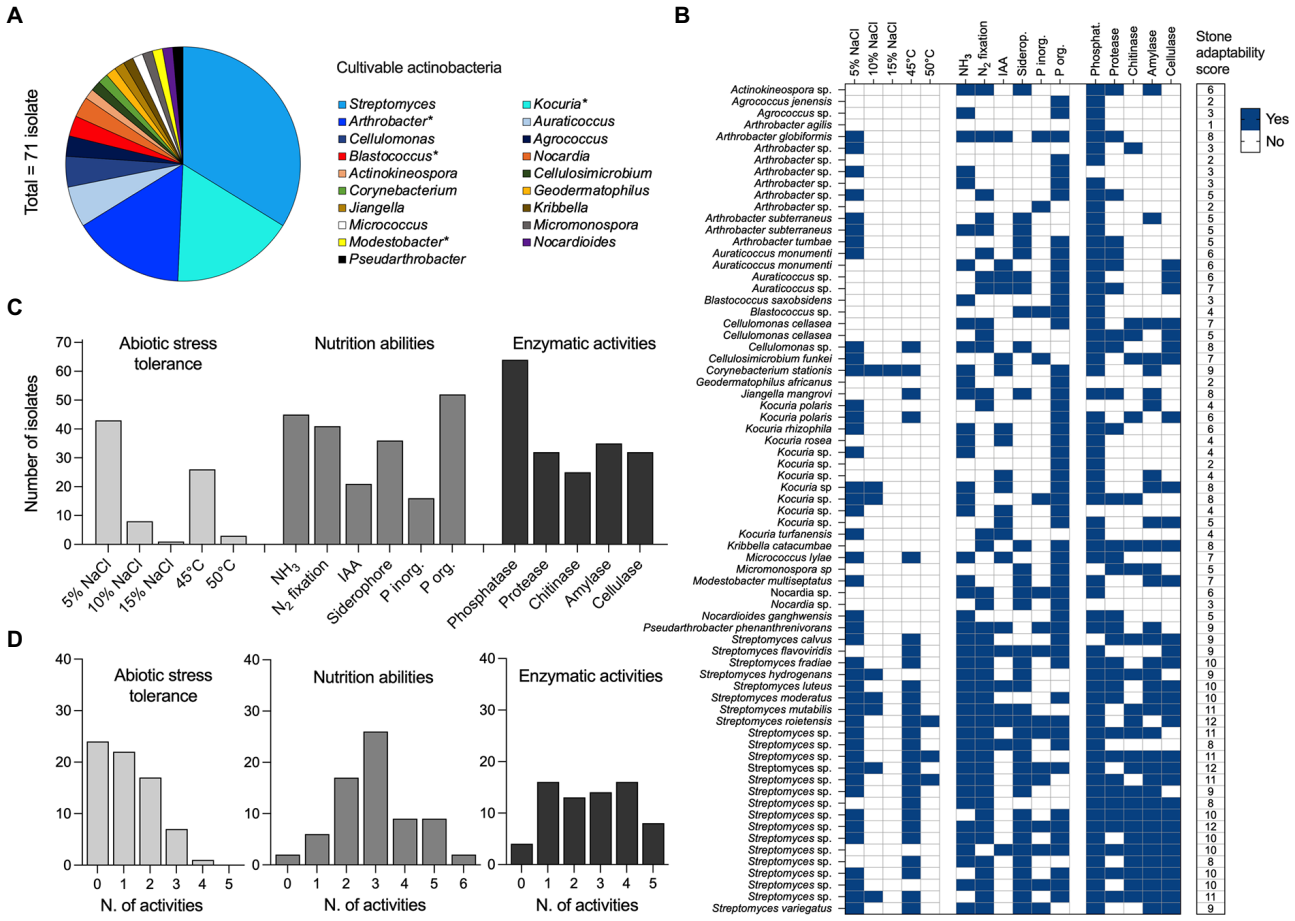
Diversity and adaptation of cultivable actinobacteria from stones. **(A)** Bacterial diversity of culturable bacterial isolates (*n* = 71) associated with stone materials was reported at the genus level. All isolates are classified within the *Actinobacteria* class. **(B)** The heat map reports the distribution of (i) abiotic stress tolerance (*n* = 5; salinity 5%, 10%, and 15% of NaCl, and temperature 45°C and 50°C), (ii) nutrition abilities (*n* = 6; NH_3_ and IAA production, fixation of N_2_, the release of siderophore and solubilization of inorganic and organic P) and (iii) enzymatic activities (*n* = 5; phosphatase, protease, chitinase, amylase, and cellulase) across the 71 bacterial isolates. The results are expressed as the presence (blue = yes) and the absence (white = no) of each activity tested *in vitro*. A final score of “stone adaptability” is also computed by summing the presence of activity across the three categories and reported in the last column; the possible maximum value of “stone adaptability” that indicates the presence of all the activity is 16. **(C)** Total number of bacterial isolates positive to the different activities, within the abiotic stress tolerance, nutrition abilities and enzymatic activities categories listed above. **(D)** Distribution of bacterial isolates across the activities’ occupancy; range 0 (no one of the activity) to a maximum for the different categories (5 for abiotic stress tolerance and enzymatic activities, and 6 for nutrition abilities).

We assessed the diversity of cultivable actinobacteria associated with stone samples. A total of 125 bacterial colonies were isolated on oligotrophic media, and 71 of them were further selected based on their growth characteristics and colony properties (color, size, pigment formation) as possible actinobacteria. ITS-PCR fingerprinting detected 28 distinct haplotypes (Supplementary Figure S6). All were identified within the *Actinobacteria* class only and encompassed three orders (*Actinomycetales*, *Geodermatophilales*, *Jiangellales*) and 14 families (*Streptomycetaceae*, *Micrococcaceae*, *Promicromonosporaceae*, *Microbacteriaceae*, *Nocardiaceae*, *Nocardioidaceae*, *Micromonosporaceae*, *Propionibacteriaceae*, *Cellulomonadaceae*, *Actinosynnemataceae*, *Geodermatophilaceae*, *Jiangellaceae*, *Brevibacteriaceae*, and *Corynebacteriaceae*). At the genus level (Figure 6A), the isolates were identified within 19 genera where *Streptomyces* was the most common (33.8%), represented by different species, including *S. variegatus*, *S. luteus*, *S. mutabilis, S. moderatus*, *S. hydrogenans*, *S. flavoviridis*, *S. roietensis*, *S. fradiae*, and *S. calvus,* along with several unclassified species (Figure 6B). The *Kocuria* genus was the second most abundant (16.9%), followed by *Arthrobacter* (15.5%), *Auraticoccus* (5.6%) and *Cellulomonas* (4.2%). The remaining strains belong to the genera *Agrococcus, Blastococcus, Nocardia*, *Actinokineospora*, *Cellulosimicrobium*, *Corynebacterium*, *Geodermatophilus*, *Jiangella*, *Kribbella*, *Micrococcus*, *Micromonospora*, *Modestobacter*, *Nocardioides*, and *Pseudarthrobacter* (Figures 6A,B).

Despite the cultivable approach allowed us to isolate bacteria belonging to the *Actinobacteria* class only, we found that the 16S rRNA gene of 17 isolates showed similarity > 97% with 6 OTUs from NGS sequencing. These OTUs accounted 1/3 of the Actinobacteria class and occupy 1.2% of relative abundance within the entire community, suggesting that the representative cultivable strains isolated could belong to the rare microbial biosphere of Tunisian stones (Pedrós-Alió, 2006).

### Cultivable actinobacteria and their capacity to survive in stone habitats

Actinobacteria isolates (*n* = 71) were tested *in vitro* for (i) their tolerance to abiotic stresses, such as salinity (5%, 10%, and 15% NaCl) and high temperature (45°C and 50°C), and (ii) their ability to solubilize/obtain nutrients and energy for their growth (Figures 6B,C; Supplementary Figure S7). Out of 71 isolates, 59.1% and 11.2% grew in the presence of 5 and 10% NaCl, respectively, while only one *Corynebacterium stations* strain grew at the highest concentration of salt tested (15% NaCl). A total of 26 isolates (36.6%) exhibited remarkable growth at 45°C; however, at the extreme condition of 50°C, only three *Streptomyces* strains were actively grown (one *Streptomyces roietensis* and two unclassified species). The mineralization of phytate (organic P) was observed in 73.2% of isolates (range, 0.001–1.99 μg/ml), with species belonging to the *Streptomyces* genus exhibiting the greatest values (Supplementary Figure S7). Sixteen isolates (22.5%) were able to solubilize inorganic phosphate (range, 0.01–0.63 μg/ml; *Arthrobacter* sp. G63, *Blastococcus* sp. G86 and *Kocuria* sp. K2 showed the highest values), while 45 isolates (63.3%) produced ammonia (range, 0.05–12.45 μg/ml; *Kocuria* sp. K2 had the highest production). As well, 41 isolates (57.7%) grew on N_2_ free medium, 21 (29.5%) produced IAA, and 36 isolates (49.2%) produced siderophore. The actinobacteria isolates were further tested *in vitro* for enzymatic activities, namely phosphatase, protease, chitinase, amylase and cellulase (Figures 6B,C and Supplementary Figure S7). The results revealed the potential of the strains to produce a wide range of hydrolytic enzymes. A total of 64 isolates (90.1%) possessed phosphatase, while 35 (49.2%) showed amylase activity, 32 (45.1%) cellulase and protease activities, and 25 (35.2%) chitinolytic activity. The “stone adaptability” potential of the isolates has been also evaluated by attributing a score based on the positive number of activities tested (minimum 0, maximum 16; see last column in Figure 6B). Bacterial isolates showed a stone adaptability score ranging from 1 to 12, with no one of the strains showing 0 or 13 (and more) activities. Notably, 45% of the strains showed multiple stone adaptations with 8–12 activities. Such high scores were observed among all cultivable *Streptomyces* (24 strains), and strains from *Arthrobacter* (2), *Corynebacterium* (1), *Cellulomonas* (1), *Kribbella* (1), *Kocuria* (1), *Jiangella* (1), and *Pseudarthrobacter* (1). Except for *Streptomyces*, all the other genera hosted isolates with a variable number of activities, ranging from two and seven; only one isolate from *Arthrobacter* genus (99.4% similarity with *Arthrobacter agilis*) showed only one activity (Figure 6B).

Finally, the antimicrobial activities of actinobacterial isolates were screened against 15 bacterial and fungal pathogenic organisms (Table 3). Sixteen (22.5%) out of 71 isolates exhibited appreciable inhibitory activity against at least one of the pathogens tested (Table 3). The active isolates were identified as *Streptomyces, Actinokineospora, Auraticoccus, Micromonospora,* and *Nocardia.* The growth inhibition was mainly observed against Gram-positive bacteria (*Staphylococcus aureus, Bacillus cereus* and *Enterococcus faecalis*; Figures 3A,B,D, 4E,F). For example, the growth of *S. aureus* and *B. cereus* was inhibited by seven and eight strains, respectively, but only one strain was effective against *E. faecalis.* Contrary, lower growth inhibition activity was observed against Gram-negative bacteria (*Escherichia fergusonii* and *Salmonella enterica*) that were inhibited only by two strains (*Actinokineospora* sp. and *Streptomyces s*p.); notably, no one of the isolates was active against *Pseudomonas aeruginosa* (Table 3A). In addition, the selected isolates showed inhibitory activity at least against one fungal pathogen (Table 3B). The bacterial isolates were mainly active against *Fusarium oxysporum* and *Aspergillus flavus* (inhibited by 8 active strains), while *Penicillium expansum* was inhibited by seven active strains, *Candida parapsilosis* and *Candida metapsilosis* by five active strains, *Alternaria alternata* and *Aspergillus niger* by four active strains, and *Cladosporium* sp. by only 3 active strains (Table 3B). As previously observed for the “stone adaptability” score, bacteria belonging to the *Streptomyces* genus were the most active and versatile isolates; for instance, isolates identified as the closest relative of *S. roietensis* and *S. variegatus* were able to inhibit the growth of seven pathogens on 15 tested (Table 3B).

**Table 3:** Biocontrol potential of bacterial isolates against pathogenic (A) bacteria and (B) fungi. Inhibitory profile of the bacterial isolates active at least against one of the pathogens tested, six bacteria and nine fungi.

Closest relative	Code	***Staphylococcus aureus***		***Enterococcus faecalis***	***E. faecalis***		***Escherichia fergusonii***	***Pseudomonas aeruginosa***		***Salmonella enterica***
**(A) Biocontrol of pathogenic bacteria**										
*Actinokineospora* sp.	G108	+		−	−		+	−		+
*Auraticoccus monumenti*	G70	−		−	+		−	−		−
*Micromonospora* sp.	G101	−		−	+		−	−		−
*Nocardia* sp.	G26	+		−	−		−	−		−
*Streptomyces hydrogenans*	G8	−		−	−		−	−		−
*Streptomyces luteus*	G16D	+		−	+		−	−		−
*Streptomyces moderatus*	G30	−		−	+		−	−		−
*Streptomyces mutabilis*	G7	−		−	−		−	−		−
*Streptomyces roietensis*	G11	−		−	−		−	−		−
*Streptomyces* sp.	G3	−		−	−		−	−		−
*Streptomyces* sp.	G4	+		−	+		−	−		−
*Streptomyces* sp.	G25	−		−	−		−	−		−
*Streptomyces* sp.	G44	+		+	+		−	−		−
*Streptomyces* sp.	G76	+		−	+		+	−		+
*Streptomyces* sp.	G93	−		−	−		−	−		−
*Streptomyces variegatus*	G45	+		−	+		−	−		−
Closest relative	Code	***Candida albicans***	***Candida metapsilosis***	***Candida parapsilosis***	***Penicillium expansum***	***Cladosporium***	***Aspergillus niger***	***Aspergillus flavus***	***Fusarium oxysporum***	***Alternaria alternata***
**(B) Biocontrol of pathogenic fungi**										
*Actinokineospora* sp.	G108	+	+	+	+	−	−	−	−	+
*Auraticoccus monumenti*	G70	−	−	−	−	−	−	−	−	−
*Micromonospora* sp.	G101	−	−	−	−	−	−	−	−	−
*Nocardia* sp.	G26	−	−	−	−	−	−	−	−	−
*Streptomyces hydrogenans*	G8	−	−	−	−	−	−	+	−	−
*Streptomyces luteus*	G16D	−	+	−	+	−	−	+	+	+
*Streptomyces moderatus*	G30	−	−	−	−	−	−	−	−	−
*Streptomyces mutabilis*	G7	−	−	−	−	−	−	−	+	−
*Streptomyces roietensis*	G11	−	−	+	+	+	+	+	+	+
*Streptomyces* sp.	G3	−	−	−	−	−	−	+	+	−
*Streptomyces* sp.	G4	−	−	−	+	−	+	+	+	+
Closest relative	Code	***Candida albicans***	***Candida metapsilosis***	***Candida parapsilosis***	***Penicillium expansum***	***Cladosporium***	***Aspergillus niger***	***Aspergillus flavus***	***Fusarium oxysporum***	***Alternaria alternata***
*Streptomyces* sp.	G25	+	−	+	+	−	−	+	+	−
*Streptomyces* sp.	G44	−	−	−	+	+	+	+	+	−
*Streptomyces* sp.	G76	−	+	+	−	−	−	−	−	−
*Streptomyces* sp.	G93	+	+	+	−	−	−	−	−	−
*Streptomyces variegatus*	G45	+	+	−	+	+	+	+	+	−

List of pathogenic bacteria tested *Staphylococcus aureus, Enterococcus faecalis, Bacillus cereus, Escherichia fergusonii, Pseudomonas aeruginosa*, *and Salmonella enterica.* Results: –, absence of growth inhibition; +, presence of growth inhibition. List of pathogenic fungi tested: *Candida albicans, Candida metapsilosis, Candida parapsilosis, Penicillium expansum, Cladosporium sp., Aspergillus niger, Aspergillus flavus, Fusarium oxysporum, Alternaria alternata.* Results: –, absence of growth inhibition; +, presence of growth inhibition.

## Discussion

Many studies have emphasized environmental samples from unexplored or underexplored natural habitats (Zhang et al., 2020). Among these habitats, stones are complex environments composed of several micro-niches that permit the proliferation of several microorganisms despite the harsh conditions resulting from low water and nutrient availability (Cockell et al., 2002; Gorbushina et al., 2002). Relatively limited information on the biodiversity of the bacterial communities of open-air stone ruins exposed to the atmosphere was previously reported, especially under the harsh environmental conditions (e.g., limited precipitations) occurring at the latitudes studied in this investigation. With this outlook, the present research has been initiated to analyze the bacterial communities of stone ruins sampled from three regions in Tunisia, each representing a distinct climate using high-throughput cultivation-independent approaches, which have been extensively applied in microbial ecology research (Sun et al., 2018), along with cultivation approach to characterize the adaptation and role of the cultivable portion of stone microbiome.

Analysis of the bacterial communities revealed that Cyanobacteria, Proteobacteria and Actinobacteria dominate the stone microbiome of Tunisia ruins, as also reported in previous study conducted in north Africa (Louati et al., 2020). Members of these bacterial phyla have specific ecological roles within the stone microbiome. For instance, Cyanobacteria support the community through photosynthesis and primary production, Proteobacteria aid biofilm development and consequently promote water retention and microbial community development, and Actinobacteria by extending their hyphae maintain a stable structure and attachment of the community (Eppard et al., 1996; Kiel and Gaylarde, 2006; Abdulla, 2009; Horath and Bachofen, 2009). In addition, the latest present high genomic GC content, complex peptidoglycan layer forming a thick cell wall, motility, spore-bearing cells, and production of pigments, all traits to over the challenging conditions of stone habitats (Mohammadipanah and Wink, 2016).

Despite the consistent presence of such groups, they were differential distributed across different climates. This result confirms the diversity pattern previous observed in other works (Meslier et al., 2018; Louati et al., 2020), in which climate significantly impacts stone microbial communities’ structure, especially if the level of water received by the community and its availability is considered. Along with climate conditions, the geochemical properties of the stone ruins also impact the stone microbiomes. Biologically available Mg, Cr, and Cu concentrations were correlated with the microbial community diversity. Most previous research on the microbiome of stone structures has focused on stone geochemistry as the primary driver of diversity and functional variation (Chimienti et al., 2016; Brewer and Fierer, 2018). Although these stones host microbial communities that were distinct by climate, several taxa that were shared in all samples were identified and can be described as the ‘core microbiome’, including Actinobacteria genera *Rubrobacter*, *Actinoplanes*, and *Blastococcus*, members of the Proteobacteria groups *Sphingomonas*, *Rubellimicrobium*, and *Craurococcus*, each of which is known for their advanced ability of adaptation to colonize extreme environments characterized by stressful conditions related to high temperature, dryness, and low nutrient availability.

To obtain more insight on the “stone adaptability” potential of the bacterial community, we characterize *in vitro* the cultivable portion of bacteria, mainly focusing on actinobacteria. As previously described, the cultivable actinobacteria encompass different genera previously isolated from stones (Laiz et al., 2003; Subramani and Sipkema, 2019), such as *Streptomyces, Kocuria, Arthrobacter Micrococcus, Pseudarthrobacter,* and *Brevibacterium.* Most of these genera are considered “rare actinobacteria” (Azman et al., 2015; Fang et al., 2019) because they are less frequently isolated than other actinobacteria, such as *Streptomyces* that also in our collection was dominant. However, the phylogeny of cultivable actinobacteria recovered from stones varied from study to study, depending on the set of media used (Essoussi et al., 2012). Cultures and isolation media that are nutritionally rich usually fail to cultivate extensive diversity of bacteria from complex natural samples (Lasudee et al., 2018).

Despite the culture-independent molecular methods are suitable for obtaining the broadest possible information on the bacterial diversity in a sample, their outcome does not consistently match those obtained by the cultivation approaches (Horath and Bachofen, 2009). For example, in our study culture-independent methods revealed the presence of *Acidimicrobiia, Thermoleophilia*, *Rubrobacteria*, and *Actinobacteria* classes, whereas culture-dependent techniques have recovered only bacteria within the *Actinobacteria* class. It is important to note that the successfully cultivable *Streptomyces* bacteria were only detected by culture-dependent technique. The differences between the results of culture-dependent and culture-independent techniques must lie in the physiological state of the microorganisms (Laiz et al., 2003). Unculturable species were probably present as quiescent vegetative forms or were lysed after prolonged dryness, lack of nutrients, or other unfavorable conditions (Laiz et al., 2003). If the requirements for the growth of a strain are met, it may be isolated whether it is abundant or not. At the same time, culture-independent methods generally detect the strains that are present above a certain threshold level, i.e., abundant strains probable to carry out most of the functions of the community (Pedrós-Alió, 2006).

Adaptation to the harsh environmental conditions prevalent in stone ruins might have led the *Actinobacteria* to produce their potential secondary metabolites and metabolic processes that are more diverse than those produced by other microorganisms (Uddin and Agsar, 2012). Among such metabolic processes, phosphorous solubilization is crucial for effectively releasing available P through solubilization and mineralization. Microbial solubilization of mineral or organic phosphates is mediated by both organic acids and enzymes, such as alkaline phosphatase (Vyas et al., 2009). Many actinobacterial isolates from the ruin stones exhibited phosphate solubilization and phytate mineralization capacities, such as *Streptomyces*, *Nocardia*, *Arthrobacter*, *Kocuria*, *Blastococcus*, and *Pseudarthrobacter*. According to other previous reports, these genera have also been described as phosphate solubilizers (Gangwar et al., 2014; Bhatti et al., 2017). Biological nitrogen fixation is the mechanism that converts atmospheric nitrogen (N_2_) to ammonia, which plants can easily use (Şahin et al., 2004; Orhan et al., 2006; Figueiredo et al., 2008). Many genera were reported in this study, such as *Arthrobacter*, *Nocardia* sp. (Metcalfe and Brown, 1957), *Auraticoccus* (Setten et al., 2013), *Pseudarthrobacter* (Fang et al., 2019), are known to fix nitrogen. Plant growth can be enhanced by the microbial production of phytohormones like IAA. The IAA influences several parameters in the plant, namely root elongation, cell division and differentiation of vascular tissues (Sousa and Olivares, 2016). In the present study, 26% of the cultivated *Streptomyces* produce IAA with an amount ranging from 19.6 to 118.9 μg/ml. Siderophores are small molecules of iron-chelating compounds produced under iron starvation by several microorganisms, including Actinobacteria (Castignetti and Smarrelli, 1986). Siderophores are valuable compounds in many possible applications; for instance, they are known to promote plant growth and inhibit plant pathogens (Ahemad and Kibret, 2014), and these molecules have been widely used in agricultural, environmental, and biomedical applications (Chaiharn et al., 2008). In the stone environment, the producers of these molecules can solubilize and uptake the limited amounts of iron or other essential metals that may be necessary for growth. In our study, actinobacteria belonging to the *Streptomyces*, *Nocardia*, *Cellulomonas*, *Auraticoccus*, *Arthrobacter*, *Kribbella*, *Jiangella*, *Modestobacter, Micromonospora*, *Streptomyces*, and *Actinokineospora* genera were able to produce siderophores. The genera *Actinokineospora* and *Auraticoccus* were not previously reported as siderophore producers. Besides the production of siderophores, another mechanism that can favor the growth on and in stone materials is the production of hydrolytic enzymes, such as cellulase, protease, amylase and chitinase. These hydrolytic enzymes participate in the degradation of organic matter that may deposit on the stone surface with dust, thus favoring substrates’ availability for microbial growth. On the other hand extracellular enzymatic profile may provide information about the other microbial communities for providing varying substrate for heterotrophic bacteria. The biochemical deteriorating effect of actinomycete depicts the involvement of extracellular enzymes secreted by them and causing degradation or deterioration of substrate. Actinomycete cause deterioration of stone by means of both physical and chemical attack which more often result synergistic in deterioration(Abdulla et al., 2008). Biochemical action of actinomycetes may lead to pitting and etching, mineral dislocation and dissolution (Abdulla, 2009). Number of extracellular hydrolytic enzymes excreted by actinomycete lead to the formation of acidic products that cause chemical changes of the material under attack ((Chen et al., 2021). The most prevalent enzymes involved in deterioration are Cellulases which catalyze hydrolysis of cellulose, break internal bonds to disrupt its crystalline structure (Abdulla, 2009). Proteases a complex group of hydrolytic enzymes, which add water across amide bonds and thus hydrolyze proteins into small peptides and amino acid (Abdulla, 2009). Most isolates examined in this study produced at least one extracellular enzyme. Actinobacteria are considered vital sources of clinically significant antibiotics (Baltz, 2007) and antagonistic compounds that can privilege these microorganisms in competition with other microorganisms for the limited recourses and niche occupation in the stones. In the natural environment, antimicrobial compounds are likely to limit the growth of competitors, thereby offering a selective advantage to the producer, in particular when nutrients become limited and the developmental program leading to spores commences (He et al., 2022). Production of bioactive compounds is typically linked to the developmental lifecycle, and antibiotics are presumably produced to safeguard the nutrient supply during developmental growth (Ali et al., 2020). Actinobacteria produce an arsenal of degradative enzymes (e.g., glycosyl hydrolases, lipases and proteases), which combined with the production of antibiotics and the ability to form desiccation resistant exospores has facilitated their success in a multitude of soil environments and sediments including those of surface stone(Heul et al., 2018). This property may be adopted due to their survival in harsh conditions where they face unusual conditions by switching on certain genes to face harsh conditions and to encounter the effects of dominant competing microorganism. Previous studies have shown that only 1%–10% of isolates from soil have antimicrobial effects *in vitro* (Gardener and Fravel, 2002; Kamilova et al., 2006), while in our collection we found 62% of the strains active against Gram-positive bacteria. It has been reported that the sensitivity of bacteria to antibiotics is variable, and Gram-positive bacteria are more sensitive than those Gram-negative (Bouchlaghem et al., 2020). The most active and multi-target isolates belong to the genus *Streptomyces.* This genus is widely recognized for its ability to synthesize numerous bioactive metabolites. Approximately 60% of the antibiotics were discovered from the *Streptomyces* genus (Bérdy, 2012). Species of this genus like *S. mutabilis* (Hamed et al., 2017), *S. hydrogenans* (Kaur and Manhas, 2014), and *S. variegates* (Canh et al., 2017) are known to be producers of effective antibiotic molecules. To our knowledge, species of *S. moderatus*, *S. roietensis* and *S. luteus* that were isolated from the ruin stones have not previously been reported as producers of antimicrobial compounds. It is also the case with the actinobacterial genus *Auraticoccus*.

Driven by the substantial overlap in the structures of newly discovered antimicrobial compounds from *Streptomyces*, researchers have begun to focus on rare actinobacteria, which are viewed as vast potential reservoirs of novel natural products (Dhakal et al., 2017). Some of the rare actinobacteria detected in this study, such as members of *Actinokineospora*, *Micromonospora*, and *Nocardia*, inhibited the growth of pathogen indicator organisms, showing their potential as antagonist strains, which should give these bacteria a competitive advantage during stone colonization: they are equipped to compete for space and resources against the other members of the community, as recently showed during the colonization of “mini-oases” plant-mediated in hyperarid desert ecosystems (Marasco et al., 2022). Also, these microorganisms should not be ignored for their potential in the research for natural products with biotechnological applications. Several researchers argue that rare actinomycetes represent a bacterial group which is highly diverse and understudied (Tiwari and Gupta, 2012). The importance of these strains is particularly demonstrated by the discovery of many effective antibacterial agents, such as gentamicin from *Micromonopsora* (Bérdy, 2005). Such potential of controlling the growth of other microorganisms is a potent competitive advantage for utilizing the few resources available on the stone material and represents an important feature to winning the competition for the colonization of such materials.

## Conclusion

Our data contribute to the knowledge of the microbial inhabitants of stone materials, their distribution and biodiversity and the environmental factors that determine the microbial community assembly on the mineral substrates of the stones. We have shown that climate and geochemical characteristics of stones are primary factors that regulate the structure of stone communities, possibly by affecting the rate of microbial colonization and restricting access to minerals and nutrients needed for growth. Our findings also provide indications on the microbial types that should be considered as potential organisms that may contribute either to the color alteration or the physical decay of stone surfaces or contribute to preserving the structure and esthetic features of such materials.

## Data availability statement

The datasets presented in this study can be found in online repositories. The names of the repository/repositories and accession number(s) can be found below: https://www.ncbi.nlm.nih.gov/bioproject/, PRJNA847910.

## Author contributions

IS, H-IO, and AM: conceived and designed the study and experiments. AM and AC: sample collection. IS: performed the experiments. IS, RM, and GT: analyzed the data. IS, RM, MG, DD, H-IO, GT, and AM: contributed reagents, materials, and analysis tools. IS: wrote the paper: GT, RM, and H-IO: revised the manuscript. All authors contributed to the article and approved the submitted version.

## Funding

This research has been conducted with the financial support of the Tunisian Ministry of Higher Education and the Erasmus+ Program of the European Union, Inter-institutional agreement between institutions from program and partner countries.

## Conflict of interest

The authors declare that the research was conducted in the absence of any commercial or financial relationships that could be construed as a potential conflict of interest.

## Publisher’s note

All claims expressed in this article are solely those of the authors and do not necessarily represent those of their affiliated organizations, or those of the publisher, the editors and the reviewers. Any product that may be evaluated in this article, or claim that may be made by its manufacturer, is not guaranteed or endorsed by the publisher.

## Footnotes

^1^ http://www.ncbi.nlm.nih.gov
