# Supplementary material for: Diversity and adaptation properties of actinobacteria associated with Tunisian stone ruins
Source: Front Microbiol. 2022 Dec 13;13:997832. doi: 10.3389/fmicb.2022.997832 (PMC9793712; doi:10.3389/fmicb.2022.997832)
Supplement: Supplementary file 1 [file Data_Sheet_1.docx]

**SUPPLEMENTARY TABLES**

**Supplementary Table S1.** Geochemical Analysis of the stone samples collected from the three different sites: ME (Mediterranean), SA (semi-arid) and AR (arid). Star (*) indicates significant differences of a certain element between the stones collected from the ruins along the aridity gradient as determined by the analysis of variance (ANOVA) test (*p* < 0.05). Arsenic (As), Plomb (Pb), Cadmium (Cd), Chrome (Cr), Copper (Cu), Iron (Fe), Zinc (Zn), Calcium (Ca), Magnesium (Mg) and Sodium (Na).

| **Samples** | **As*** | **Pb** | **Cd** | **Cr*** | **Cu*** | **Fe*** | **Zn** | **Ca** | **Mg*** | **Na*** | **K** |
| --- | --- | --- | --- | --- | --- | --- | --- | --- | --- | --- | --- |
| ME-1 | 0.06 | 0.06 | 0.28 | 0.034 | 0.13 | 0.98 | 1.18 | 241.81 | 230.3 | 15.67 | 74.07 |
| ME-2 | 0.05 | 0.05 | 0.26 | 0.038 | 0.12 | 0.88 | 1.15 | 264.51 | 244.6 | 16.75 | 78.15 |
| ME-3 | 0.05 | 0.06 | 0.29 | 0.036 | 0.12 | 0.86 | 1.12 | 286.75 | 254.25 | 15.55 | 84.19 |
| SA-1 | 0.07 | 0.06 | 0.28 | 0.048 | 0.15 | 1.10 | 1.28 | 272.48 | 265.5 | 13.22 | 76.42 |
| SA-2 | 0.06 | 0.06 | 0.3 | 0.05 | 0.16 | 1.10 | 1.3 | 270.58 | 265.69 | 13.89 | 75.39 |
| SA-3 | 0.07 | 0.05 | 0.27 | 0.04 | 0.15 | 1.10 | 1.12 | 271.54 | 264.7 | 12.90 | 76.20 |
| AR-1 | 0.05 | 0.05 | 0.31 | 0.053 | 0.17 | 1.06 | 0.98 | 288.64 | 244.8 | 13.74 | 82.35 |
| AR-2 | 0.04 | 0.06 | 0.31 | 0.058 | 0.16 | 1.06 | 0.98 | 256.30 | 242.6 | 12.88 | 83.15 |
| AR-3 | 0.05 | 0.05 | 0.29 | 0.049 | 0.15 | 1.1 | 1.15 | 269.25 | 238.5 | 13.44 | 84.12 |

**Supplementary Table S2.** Relationship between alphadiversity indices (richness, Shannon diversity and Simpson diversity) and precipitation (mm), latitude (m) and geochemistry (Euclidean distance). Results of linear regressions are reported. In bold are indicated the significant *p*-values (*p* < 0.05).

| **Factor** |  | **Richness** | **Shannon diversity** | **Simpson diversity** |
| --- | --- | --- | --- | --- |
| Precipitation | R^2^ | 0.4855 | 0.2969 | 0.4043 |
|  | F | 6.605 | 2.955 | 4.752 |
|  | DFn, DFd | 1,7 | 1,7 | 1,7 |
|  | *p*-value | **0.0370** | 0.1293 | 0.0657 |
| Altitude | R^2^ | 0.07316 | 0.3102 | 0.1973 |
|  | F | 0.5526 | 3.148 | 1.721 |
|  | DFn, DFd | 1,7 | 1,7 | 1,7 |
|  | *p*-value | 0.4815 | 0.1193 | 0.2309 |
| Geochemistry |  | 0.2355 | 0.2349 | 0.3147 |
|  |  | 10.47 | 10.44 | 15.61 |
|  |  | 1,34 | 1,34 | 1,34 |
|  |  | **0.0027** | **0.0027** | **0.0004** |

**Supplementary Table S3.** Taxonomic affiliation of the most abundant OTUs detected across the different clusters (Figure 4).

| **Cluster** | **OUT_ID** | **Phylum** | **Class** | **Order** | **Family** | **Genus** |
| --- | --- | --- | --- | --- | --- | --- |
| 1 | Otu2 | Cyanobacteria | Oxyphotobacteria | Nostocales | Chroococcidiopsaceae |  |
| 1 | Otu28 | Chloroflexi | Chloroflexia | Thermomicrobiales | JG30-KF-CM45 |  |
| 1 | Otu41 | Proteobacteria | Alphaproteobacteria | Acetobacterales | Acetobacteraceae | Acidiphilium |
| 1 | Otu45 | Proteobacteria | Alphaproteobacteria | Rhodobacterales | Rhodobacteraceae | Rubellimicrobium |
| 1 | Otu46 | Actinobacteria | Actinobacteria | Kineosporiales | Kineosporiaceae | Quadrisphaera |
| 2 | Otu20 | Actinobacteria | Thermoleophilia | Solirubrobacterales | Solirubrobacteraceae | Solirubrobacter |
| 2 | Otu27 | Proteobacteria | Gammaproteobacteria | Pseudomonadales | Moraxellaceae | Acinetobacter |
| 2 | Otu31 | Acidobacteria | Blastocatellia (Subgroup 4) | Pyrinomonadales | Pyrinomonadaceae | RB41 |
| 2 | Otu556 | Proteobacteria | Gammaproteobacteria | Enterobacteriales | Enterobacteriaceae | Pantoea |
| 2 | Otu82 | Chloroflexi | Gitt-GS-136 |  |  |  |
| 3 | Otu11 | Cyanobacteria | Oxyphotobacteria | Nostocales | Chroococcidiopsaceae |  |
| 3 | Otu12 | Cyanobacteria | Oxyphotobacteria | Nostocales | Cyanobacteriaceae |  |
| 3 | Otu16 | Cyanobacteria | Oxyphotobacteria | Leptolyngbyales | Leptolyngbyaceae | Leptolyngbya |
| 3 | Otu18 | Cyanobacteria | Oxyphotobacteria | Thermosynechococcales | Thermosynechococcaceae | Loriellopsis LF-B5 |
| 3 | Otu19 | Cyanobacteria | Oxyphotobacteria | Nostocales | Chroococcidiopsaceae |  |
| 3 | Otu40 | Proteobacteria | Gammaproteobacteria | Oceanospirillales | Halomonadaceae | Carnimonas |
| 4 | Otu13 | Cyanobacteria | Oxyphotobacteria | Nostocales | Chroococcidiopsaceae | Aliterella CENA595 |
| 4 | Otu15 | Proteobacteria | Alphaproteobacteria | Rickettsiales | Anaplasmataceae | Wolbachia |
| 4 | Otu4 | Cyanobacteria | Oxyphotobacteria | Nostocales |  |  |
| 4 | Otu44 | Cyanobacteria | Oxyphotobacteria | Nostocales |  |  |
| 4 | Otu49 | Actinobacteria | Actinobacteria | Micromonosporales | Micromonosporaceae | Actinoplanes |
| 4 | Otu5 | Cyanobacteria | Oxyphotobacteria | Nostocales | Chroococcidiopsaceae | Chroococcidiopsis |
| 5 | Otu25 | Actinobacteria | Rubrobacteria | Rubrobacterales | Rubrobacteriaceae | Rubrobacter |
| 5 | Otu26 | Proteobacteria | Alphaproteobacteria | Acetobacterales | Acetobacteraceae | Craurococcus |
| 5 | Otu29 | Actinobacteria | Actinobacteria | Frankiales |  |  |
| 5 | Otu30 | Proteobacteria | Alphaproteobacteria | Sphingomonadales | Sphingomonadaceae | Sphingomonas |
| 5 | Otu33 | Acidobacteria | Blastocatellia (Subgroup 4) | Blastocatellales | Blastocatellaceae | Blastocatella |
| 5 | Otu34 | Deinococcus-Thermus | Deinococci | Deinococcales | Trueperaceae | Truepera |
| 5 | Otu35 | Proteobacteria | Alphaproteobacteria | Sphingomonadales | Sphingomonadaceae |  |
| 5 | Otu36 | Cyanobacteria | Oxyphotobacteria | Nostocales | Chroococcidiopsaceae | Aliterella CENA595 |
| 5 | Otu53 | Actinobacteria | Actinobacteria | Frankiales |  |  |
| 5 | Otu63 | Proteobacteria | Alphaproteobacteria | Acetobacterales | Acetobacteraceae | Craurococcus |
| 5 | Otu69 | Actinobacteria | Actinobacteria | Frankiales | Geodermatophilaceae | Blastococcus |
| 5 | Otu71 | Acidobacteria | Blastocatellia (Subgroup 4) | Pyrinomonadales | Pyrinomonadaceae | RB41 |
| 5 | Otu8 | Cyanobacteria | Oxyphotobacteria | Nostocales | Chroococcidiopsaceae |  |
| 6 | Otu1 | Cyanobacteria | Oxyphotobacteria | Nostocales | Chroococcidiopsaceae | Aliterella CENA595 |
| 6 | Otu115 | Cyanobacteria | Oxyphotobacteria | Nostocales | Chroococcidiopsaceae | Aliterella CENA595 |
| 6 | Otu14 | Cyanobacteria | Oxyphotobacteria | Nostocales | Chroococcidiopsaceae | Chroococcidiopsis CC1 |
| 6 | Otu3 | Cyanobacteria | Oxyphotobacteria | Nostocales | Chroococcidiopsaceae |  |
| 6 | Otu39 | Cyanobacteria | Oxyphotobacteria | Nostocales | Chroococcidiopsaceae |  |

**SUPPLEMENTARY FIGURES**

**Supplementary Figure S1.** Map representing the Köppen-Geiger climate classification of Tunisia. The three sampling locations are indicated with red placemarks. KMZ for Google Earth (high res) is available at http://koeppen-geiger.vu-wien.ac.at/present.htm.


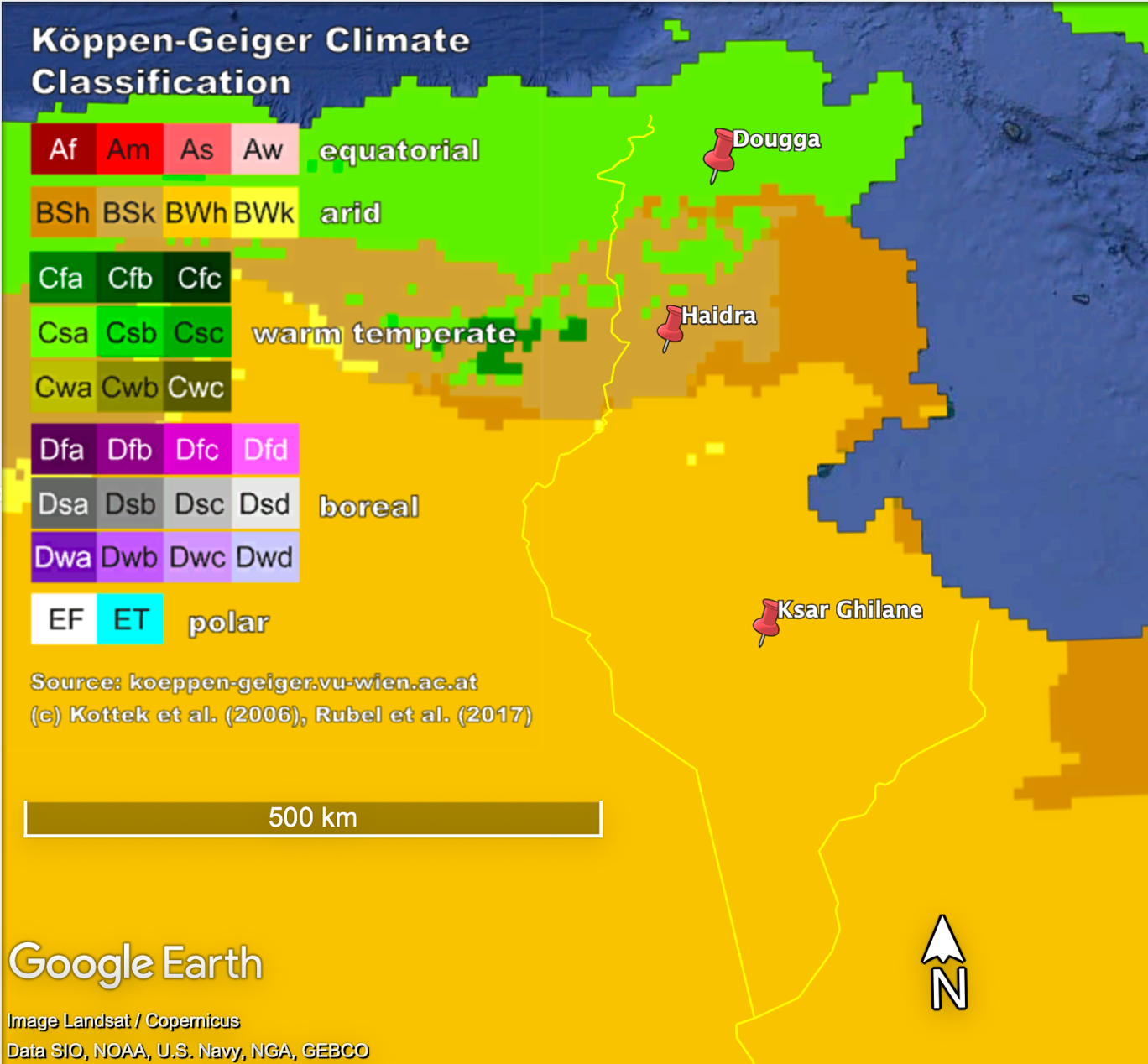


**Supplementary Figure S2.** The rarefaction curves are based on the bacterial 16S rRNA gene reads from stones sampled along Mediterranean, semi-arid and arid climates. The number of reads (sequencing depth) is reported on the x-axis, while the number of OTUs (richness) is indicated on the y-axis.

**Supplementary Figure S3.** (**A**) Canonical analysis of principal coordinates (CAP) of the elements’ composition of stones collected from the three ruins along the aridity transect. CAP cross-validation, 89% (8/9 samples); number of permutations, 999; delta_1^2, 0.97522, *p* = 0.023. (**B**) Pearson correlations of element variables are shown within the canonical axes. The data set is described in Supplementary Table S1; Arsenic (As), Plomb (Pb), Cadmium (Cd), Chrome (Cr), Copper (Cu), Iron (Fe), Zinc (Zn), Calcium (Ca), Magnesium (Mg) and Sodium (Na).

**Supporting Figure S4.** Relative abundance of bacterial classes associated with stones collected from ruins across the three climates, namely Mediterranean (ME), semiarid (SA) and arid (AR). Values are reported in percentage (*n* = 3). Results of Tukey’s multiple comparisons test are indicated by stars: *, p<0.5; **, *p*<0.01.

**Supplementary Figure S5.** Linear discriminant analysis (LDA) scores of differentially abundant taxa in stone samples. The LDA score indicates the effect size and ranking of each differentially abundant taxon.


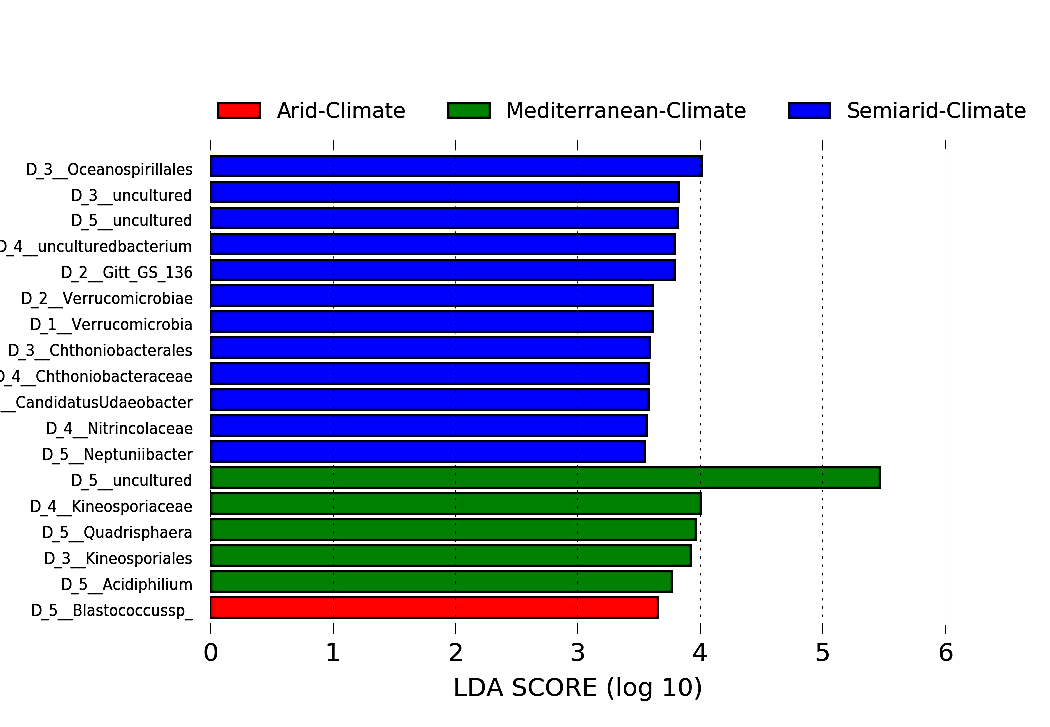


**Supplementary Figure S6.** (**A**) Phylogenetic analyses of actinobacterial isolates based on 16S rRNA gene partial sequences. Branch length support was evaluated using nonparametric bootstrap of 1000 data sets using MEGA. (**B**) Results of the amplified internal transcribed spacers 16S–23S rDNA are also reported: ITS-PCR analysis; M1: 1 kb; M2: 100 bp.


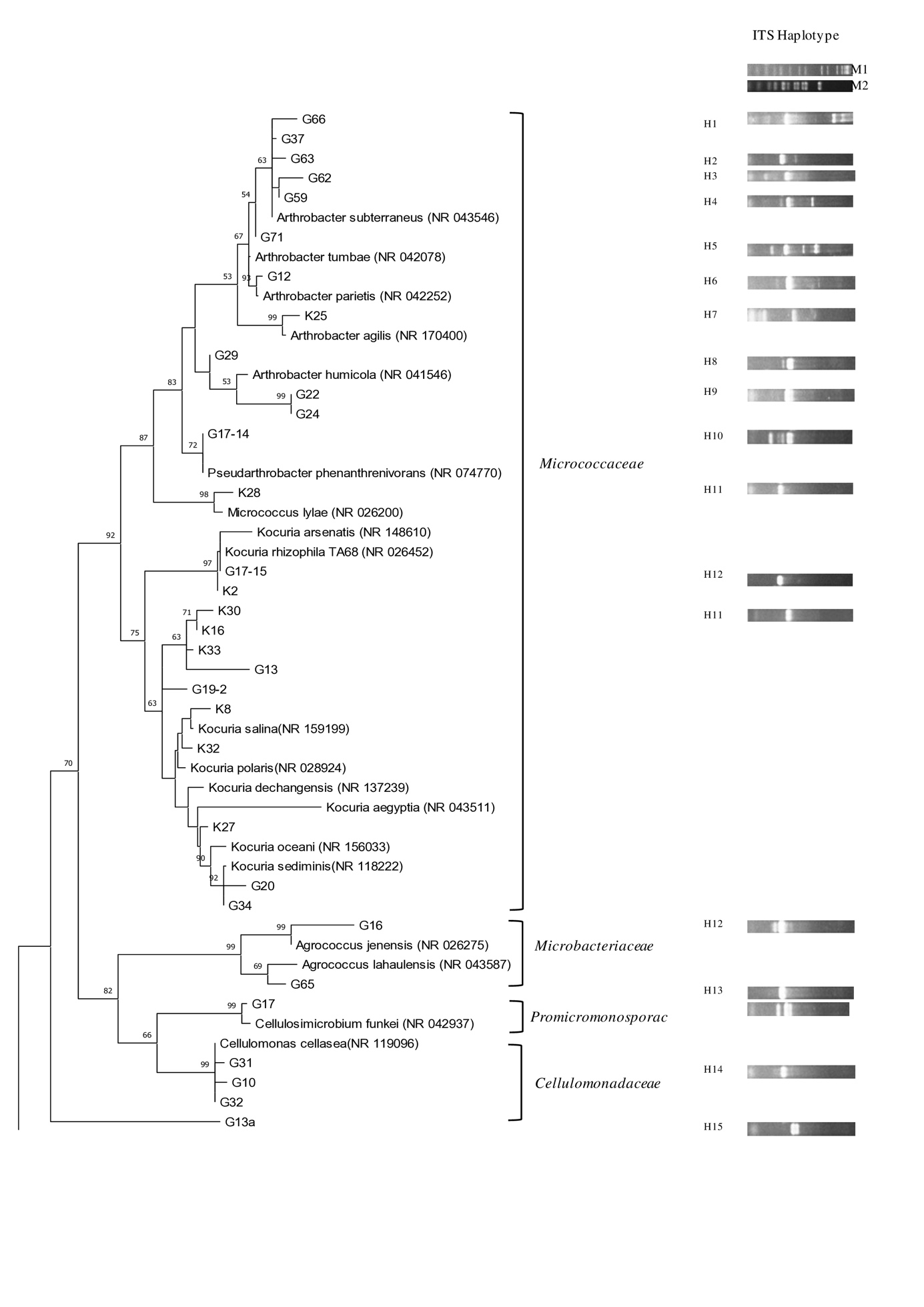


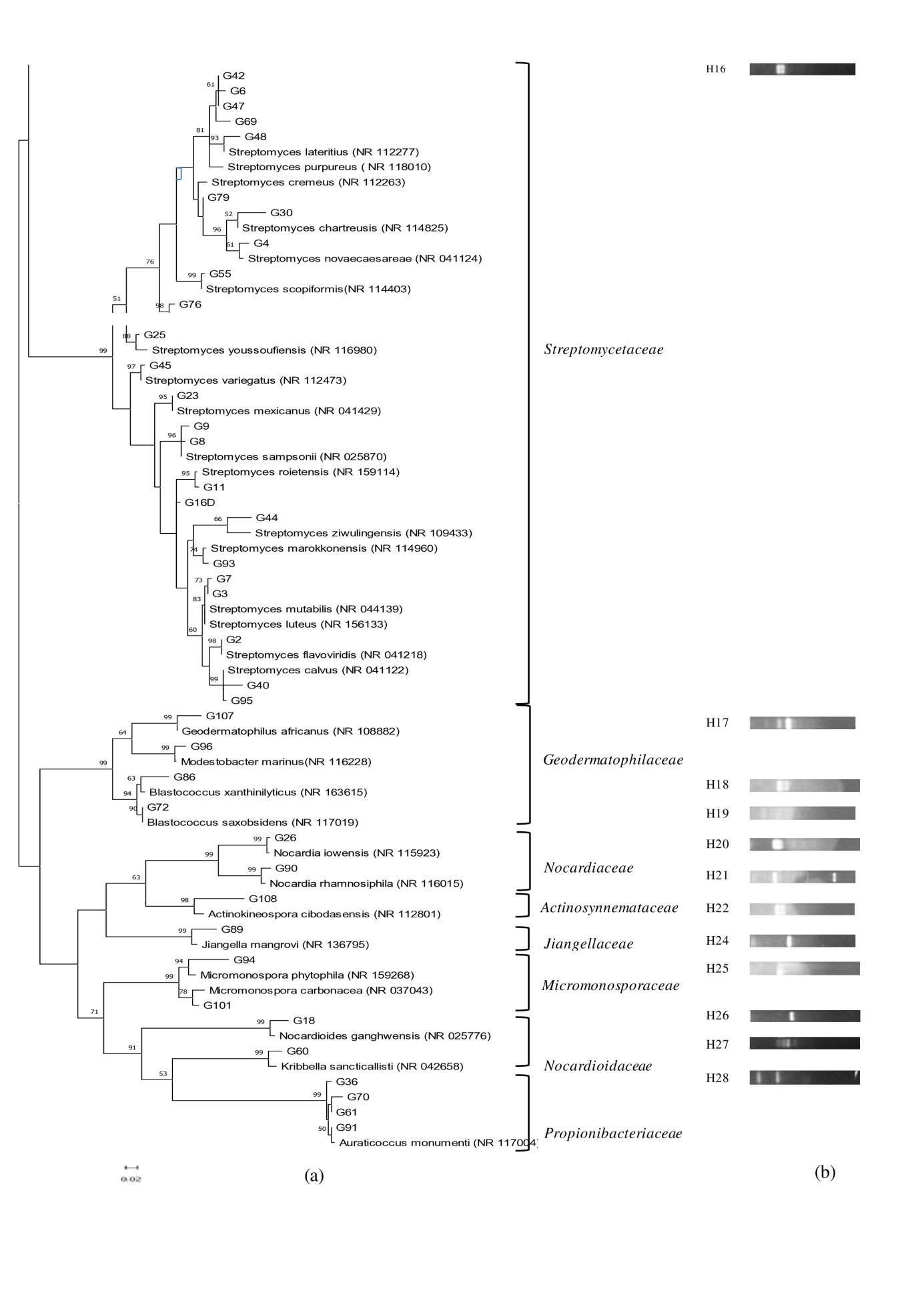


**Supplementary Figure S7.** Quantitative results of the *in vitro* tests that were performed to evaluate ammonia (NH_3_) production, solubilization of inorganic and organic phosphate (P), phosphatase activity (phosp.) and indole-3-acetic acid (IAA) production. Values are expressed as µg/ml.
